# GM‐CSF and IL‐7 fusion cytokine engineered tumor vaccine generates long‐term Th‐17 memory cells and increases overall survival in aged syngeneic mouse models of glioblastoma

**DOI:** 10.1111/acel.13864

**Published:** 2023-05-11

**Authors:** Jack M. Shireman, Nikita Gonugunta, Lei Zhao, Akshita Pattnaik, Emily Distler, Skyler Her, Xiaohu Wang, Rahul Das, Jaques Galipeau, Mahua Dey

**Affiliations:** ^1^ Department of Neurosurgery University of Wisconsin School of Medicine & Public Health, UW Carbone Cancer Center, Madison Wisconsin USA; ^2^ Department of Medicine, Division of Hematology and Oncology University of Wisconsin School of Medicine & Public Health, UW Carbone Cancer Center, Madison Wisconsin USA

**Keywords:** Aging, immunology, cytokines, GBM, senescence, T cell

## Abstract

Age‐related immune dysfunctions, such as decreased T‐cell output, are closely related to pathologies like cancers and lack of vaccine efficacy among the elderly. Engineered fusokine, GIFT‐7, a fusion of interleukin 7 (IL‐7) and GM‐CSF, can reverse aging‐related lymphoid organ atrophy. We generated a GIFT‐7 fusokine tumor vaccine and employed it in aged syngeneic mouse models of glioblastoma and found that peripheral vaccination with GIFT‐7TVax resulted in thymic regeneration and generated durable long‐term antitumor immunity specifically in aged mice. Global cytokine analysis showed increased pro‐inflammatory cytokines including IL‐1β in the vaccinated group that resulted in hyperactivation of dendritic cells. In addition, GIFT‐7 vaccination resulted in increased T‐cell trafficking to the brain and robust Th‐17 long‐term effector memory T‐cell formation. TCR‐seq analysis showed increased productive frequency among detected rearrangements within the vaccinated group. Overall, our data demonstrate that aging immune system can be therapeutically augmented to generate lasting antitumor immunity.

## Graphical abstract

Peripheral vaccination with the GIFT‐7 fusokine induces thymic regeneration, dendritic cell hyper‐activation, and T‐cell trafficking to the brain. Formation of Th‐17 based effector immunity results in rejection of contralateral intracranial tumor re‐challenge and demonstrates the effectiveness of augmenting the aged immune system with peripheral vaccination.

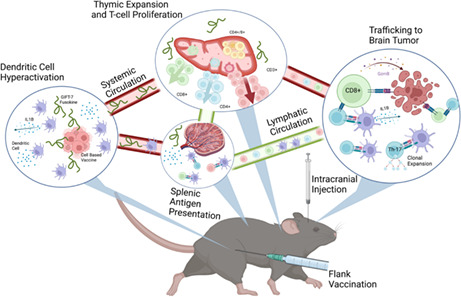

## 1 INTRODUCTION

Biological aging results from the accumulation of a multitude of cellular changes over time resulting in loss of physiological homeostasis and deterioration of various biological systems, including the immune system (Harman, 2001). Together these changes reshape physiological and immune landscapes and lead to increased vulnerability to diseases (Mogilenko et al., 2022). Aging is often the prime risk factor for many severe disorders including cancer (Berger et al., 2005; White et al., 2014), cognitive impairments (Murman, 2015; Snowdon & Study, 2003), movement disorders (Lamb et al., 2016; Mahant & Stacy, 2001), and cardiovascular disease (Aronow, 2006; Steenman & Lande, 2017). Aging‐related immune senescence limits the immune system's ability to both recognize and respond to foreign antigens or endogenous cellular distress (Boraschi et al., 2013). Aging is associated with declines in circulating T‐cell, antigen presentation, apoptotic cell clearance, as well as thymic atrophy resulting in decreased vaccine efficacy in the elderly (Aiello et al., 2019; Cepeda & Griffith, 2018; Effros, 2004; Foster et al., 2011; Gruver et al., 2007; Hsieh et al., 2015; Kanesvaran et al., 2018; Ongrádi et al., 2009; Palmer et al., 2018; Weiskopf et al., 2009; Yousefzadeh et al., 2021). Critically, not all immune cells are uniformly sensitive to aging resulting in a differential impact across several different immune cell compartments (Huang et al., 2021; Mogilenko et al., 2022; Weiskopf et al., 2009). Emerging data from the elderly, including data from our own laboratory, suggest that one of the most consequential age‐related immune dysfunctions happens within the T‐cell compartment (Effros et al., 2005; Elyahu et al., 2019; González‐Bermúdez et al., 2022; Henson et al., 2012; Huff et al., 2019, 2021; Li et al., 2019; Quinn et al., 2018; Yi et al., 2019).

Glioblastoma (GBM IDHwt), a 100% lethal primary brain cancer, is predominately a disease of older adults with a median age of diagnosis of 64 years (Ostrom et al., 2022). GBM IDHwt profoundly influences the host immune system by inducing local and systemic immune cell dysfunction (Alban et al., 2018; Chen et al., 2020; Close et al., 2020; DeCordova et al., 2020; Razavi et al., 2016); thus, there has been long‐standing interest in therapeutic manipulation of GBM IDHwt's influence on the immune system using immunotherapy. Recent studies point to a broad spectrum of T‐cell dysfunction within the tumor microenvironment that renders the durable therapeutic benefit of immunotherapy ineffective (Dunn et al., 2020; Woroniecka, Chongsathidkiet, et al., 2018; Woroniecka, Rhodin, et al., 2018). Effector arm insufficiency, characterized by CD8+ T cell dysfunction, tolerance, anergy, exhaustion, and senescence, is a hallmark of inadequate immune response against GBM IDHwt (Huff et al., 2021; Mohme et al., 2018; Woroniecka, Chongsathidkiet, et al., 2018).

Fusokines, which are engineered through the fusion of two separate and unrelated cytokines, are not bound to physiological regulation and can pharmacologically impel the clustering of unrelated but activated cytokine receptors together (Williams & Galipeau, 2011). This can result in transducing unique and supraphysiological signals that ultimately confer novel biological effects (Deng et al., 2014; Penafuerte & Galipeau, 2012; Rafei & Galipeau, 2010). GIFT (granulocyte‐macrophage colony‐stimulating factor and IL fusion transgenes) fusokines can modulate immune response, particularly in cancer and autoimmune conditions (Williams & Galipeau, 2011). GIFT‐7 is a fusokine combining the domains of both IL‐7 and GM‐CSF with a non‐biological linker (Hsieh et al., 2015). IL‐7 is critical to the development, proliferation, and survival of T‐cell in the thymus and has been explored as a supplemental therapeutic option to combat effector arm insufficiency with little clinical success (Conlon et al., 1989; Peschon et al., 1994). Novel research demonstrated that IL‐7 and its interaction with its cognate receptor (IL‐7rα) are tightly regulated biologically (Fry & Mackall, 2005; Winer et al., 2022). This tight physiological regulation necessitates supra‐therapeutic doses of the cytokine to observe a measurable thymic response, which is not achievable in vivo, limiting clinical effectiveness (Fry & Mackall, 2002; Muthukumar et al., 2004). Fusokines can aid in this therapeutic challenge because of their ability to bypass typical physiological regulation plathways (Hsieh et al., 2015; Rafei & Galipeau, 2010; Williams & Galipeau, 2011). For example, GIFT‐7 has been shown in aged mice to combat age‐induced thymic involution resulting in robust production of thymic precursor T‐cell (Hsieh et al., 2015). Mice supplemented with systemic GIFT‐7 demonstrated thymic cortical hyperplasia and responded with increased CD8+ viral specific T‐cell to a cytomegalovirus infection (Hsieh et al., 2015).

In this study, we transfected mouse syngeneic glioma cells (GL261 and CT2A) to produce GIFT‐7 and then used the transfected cells both with and without radiation treatment as a peripherally administered tumor vaccine. We found that peripheral GIFT‐7 tumor vaccine (GIFT‐7TVax) significantly increased overall survival (OS) in GL261 and CT2A aged, but not young, mice with intracranial tumor. More importantly, all the long‐term survivor (LTS) mice rejected contralateral intra‐cranial tumor re‐challenge. GIFT‐7TVax induced thymic regeneration in aged mice resulting in increased T‐cell trafficking into the brain tumor microenvironment. The GIFT‐7TVax‐induced antitumor immune response was mediated by NLRP3‐positive IL‐1β producing hyperactivated dendritic cells (DCs). GIFT‐7 vaccination limited T‐cell exhaustion and global T‐cell receptor (TCR) sequencing showed that GIFT‐7TVax resulted in focused expansion of tumor‐related TCR repertoires. GIFT‐7TVax LTS mice generated durable long‐term CD4+ Th‐17 memory T‐cells. In a clinically relevant model, aged mice vaccinated with irradiated tumor vaccine, derived from irradiated GIFT‐7 transfected glioma cells, cleared intracranial tumor implantation 100% of the time. Furthermore, vaccination with GIFT‐7TVax post‐tumor implantation resulted in the clearance of intracranial tumor in >50% of mice. These findings show that in the context of a very aggressive brain cancer, a tumor vaccine incorporating GIFT‐7 can combat the effects of aging on the T‐cell compartment and boost the efficacy of DCs resulting in a durable long‐lasting antitumor response. Thus, GIFT‐7TVax can be an effective novel tumor vaccine not only for GBM IDHwt but also for other cancers of older age.

## 2 RESULTS

### 2.1 GIFT‐7 tumor vaccine increases OS in aged mice with glioma

**FIGURE 1:**
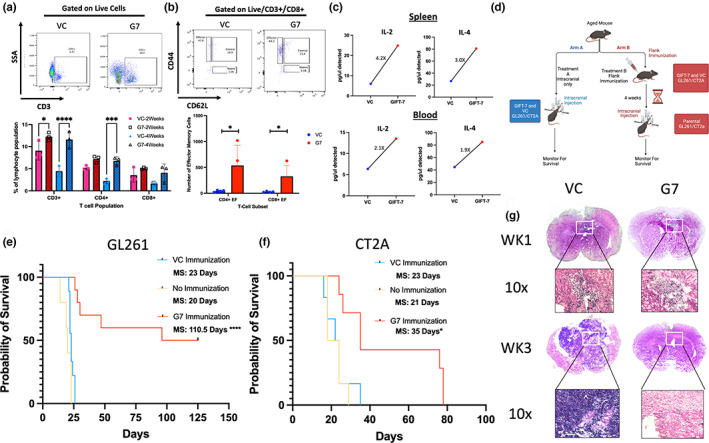
*GIFT‐7 tumor vaccine increases overall survival in aged mice with glioma*. (a) Gating and bar graph quantification for percent of live/CD3+, live/CD3+/CD4+, and live/CD3+/CD8+ cells isolated from PBMC compartment of both VC and GIFT‐7 vaccinated mice 2 and 4 weeks post‐flank vaccination implantation *n* = 3. (b) Gating and quantification of the percent of live/CD3+/CD4+/CD44+/CD62L (low) and live/CD3+/CD8+/CD44+/CD62L (low) within mouse PBMC compartment isolated 4 weeks post‐VC or GIFT‐7 vaccination *n* = 3. (c) Quantity of cytokine detected in pg/ul of conditioned media from the spleen and PBMC compartment of VC and GIFT‐7 mice 1 week post‐intracranial tumor implantation *n* = 3. (d) Experimental schematic depicting the process of both standard (intracranial only) and vaccination (VC or GIFT‐7 vaccination followed by intracranial injection) in vivo survival experiments. (e) Kaplan–Meyer survival plot of GIFT‐7 peripheral vaccination using GL261 tumor cells in aged mice across no immunization (yellow), VC immunization (blue), and GIFT‐7 immunization (red). (f) Kaplan–Meyer survival plot of GIFT‐7 peripheral vaccination using CT2A tumor cells in aged mice across no immunization (yellow), VC immunization (blue), and GIFT‐7 immunization (red). (g) Immunohistochemistry demonstrating tumor sizes 1 week and 3 weeks post‐intracranial implantation of GL261 cells in aged mice at both 2× and 10× microscopic resolution. Data are presented as mean +/− SD. Dots in bar graphs (A/B) depict individual mice, (C) depicts average cytokine reading from a biological triplicate and technical duplicate. Statistical significance was determined by corrected ANOVA (A/B/E/F) *p < 0.05, ***p < 0.001, ****p < 0.0001.

Since GIFT‐7 has been shown to reconstitute the aging thymus and increase virus‐specific effector T‐cells (Hsieh et al., 2015), we first sought to understand the impact of the GIFT‐7 fusokine locally on the intracranial tumor growth and antitumor immune response in an aged (52 weeks or above) syngeneic mouse model of glioma. To accomplish this, we transfected mouse glioblastoma lines GL261 and CT2A with GIFT‐7 plasmid allowing for endogenous production of the fusokine by the glioma cells (GL261_GIFT‐7_ vs GL261_VC_: GM‐CSF: 250 absorbance units (AU) vs. 5.69 AU *p* < 0.0001; GL261_GIFT‐7_ vs GL261_VC_: IL‐7: 1056.66 AU vs. 0 AU *p* < 0.0001) (Figure S1a). GL261 cells transfected with GIFT‐7 (GL261_GIFT‐7_) and VC (GL261_vc_) were then injected intracranially into the brains of C57/BL6 mice which were monitored for OS. No statistically significant difference in the OS was seen between the two groups (median survival (MS) VC 20 days Vs GIFT‐7 22 days) (Figure S1b). We hypothesized that the locally produced GIFT‐7 may have limited systemic presence due to the blood–brain barrier (BBB), we tested this hypothesis by generating a peripheral tumor by implanting 1,000,000 GL261_GIFT‐7_ and GL261_vc_ in the flank of C57/BL6 mice. We observed significantly smaller flank tumors in mice with GL261_GIFT‐7_ tumor cells compared to GL261_vc_ (Figure S1c). In this flank tumor model, analysis of the peripheral blood at 2 weeks and 4 weeks post‐injection showed significantly higher numbers of overall CD3+ and CD4+ T‐cells in the GL261_GIFT‐7_ group compared to GL261_vc_ (VC vs GIFT‐7 2 weeks: CD3+: 9.07% Vs 12.27% *p* < 0.05; 4 weeks: CD3+: 4.4% Vs 11.6% *p* < 0.0001; CD4+: 2.2% Vs 6.8% *p* < 0.001) (Figure 1a). T‐cell phenotype analysis showed significantly higher number of effector memory T‐cells at 4 weeks after flank implantation of GL261_GIFT‐7_ compared to GL261_vc_ (VC vs GIFT‐7: Effector Memory CD4+: 43 cells vs 537 cells *p* < 0.05; Effector Memory CD8+: 28 cells vs 328 cells *p* < 0.05) (Figure 1b). A global cytokine screen conducted at a similar timeframe also concluded that two cytokines involved in the formation and maintenance of immunological memory, IL‐2 and IL‐4 (Bosco et al., 1990; Raeber et al., 2018), were increased within both the spleen and blood of mice receiving GL261_GIFT‐7_ (Spleen IL‐2 4.2x, Spleen IL‐4 3.0×, Blood IL‐2 2.1×, Blood IL‐4 1.9×) (Figure 1c). Given the significant difference in flank tumor growth between the treatment groups and the increase in overall T‐cells, specifically effector memory T‐cells present in the peripheral blood of mice with GL261_GIFT‐7_ flanks, we wanted to understand whether the peripheral antitumor immune response was reflected in the intracranial compartment post‐flank tumor implantation. To this end, we pivoted to a peripheral vaccination model where aged mice were vaccinated in the flank using only 250,000 GL261_GIFT‐7_/GL261_vc_ cells and CT2A_VC_/CT2A_GIFT‐7_ cells, in a true vaccine format this smaller number of cells does not form a tumor mass in the flank, they function solely as a source of tumor antigen and GIFT‐7 in the periphery. Following vaccination, we waited 4 weeks and then proceeded with intracranial tumor implantation using the unmodified respective parental cell lines. To delineate the effect of peripheral immunization with VC tumor cells on the intracranial tumor, we also had a group of mice that received only intracranial tumor without any prior vaccination, and monitored all the groups for OS (Figure 1d). In both the GL261 and CT2A cell line, groups vaccinated with GIFT‐7‐modified tumor cells (GIFT‐7TVax) demonstrated a significantly increased OS when compared to VC group (MS: 23 days vs 35 days CT2A_VC_ vs GIFT‐7TVax *p* < 0.05; MS: 23 days vs 110.5 days GL261_VC_ vs GIFT‐7TVax *p* < 0.0001) (Figure 1e,f). In both the GL261_vc_ and CT2A_vc_ peripheral vaccination groups, there was no statistically significant change in OS when compared to the unvaccinated group, indicating no peripheral vaccination effect was present when unmodified cells were given. (MS: 23 days Vs 21 days CT2A_VC_ vs. no vaccination *p* = NS; MS: 23 days Vs 20 days GL261_VC_ vs no vaccination *p* = NS) (Figure 1e,f). More importantly, in the GL261 model, 50% of mice receiving GIFT‐7TVax became LTS lasting 125 days post‐intracranial tumor implantation, demonstrating full tumor clearance (Figure 1e). H & E staining of the brains of mice 1 week and 3 weeks post‐intracranial tumor implantation from the GL261_VC_ and GIFT‐7TVax groups showed smaller tumor volume in the GIFT‐7TVax group at 1 week and complete clearance of the tumor by 3 weeks (Figure 1g). To delineate the age‐specific effect of GIFT‐7, we repeated the tumor vaccination strategy in 6‐ to 8‐week‐old mice, which is the current conventional age of mice used for glioma rodent models (Dutta & Sengupta, 2016; Jackson et al., 2017; Ladomersky et al., 2016). Interestingly, in the younger mouse model, peripheral vaccination with just tumor cells significantly increased OS compared to the unvaccinated group (MS, no vaccination vs vaccination: 23 days vs ND) and GIFT‐7TVax had no additional survival advantage compared to the VC vaccine group (Figure S1d). Taken together, these findings show that in an aged mouse model of glioma, peripheral tumor vaccine in combination with GIFT‐7 can significantly slow the growth of intracranial tumor in a less immunogenic CT2A model (Gómez‐Oliva et al., 2021; Haddad et al., 2021) and can completely eradicate intracranial tumor in 50% of mice in the more immunogenic GL261 model (Gómez‐Oliva et al., 2021; Haddad et al., 2021).

### 2.2 GIFT‐7 tumor vaccine increases circulating and intratumoral T‐cell

**FIGURE 2:**
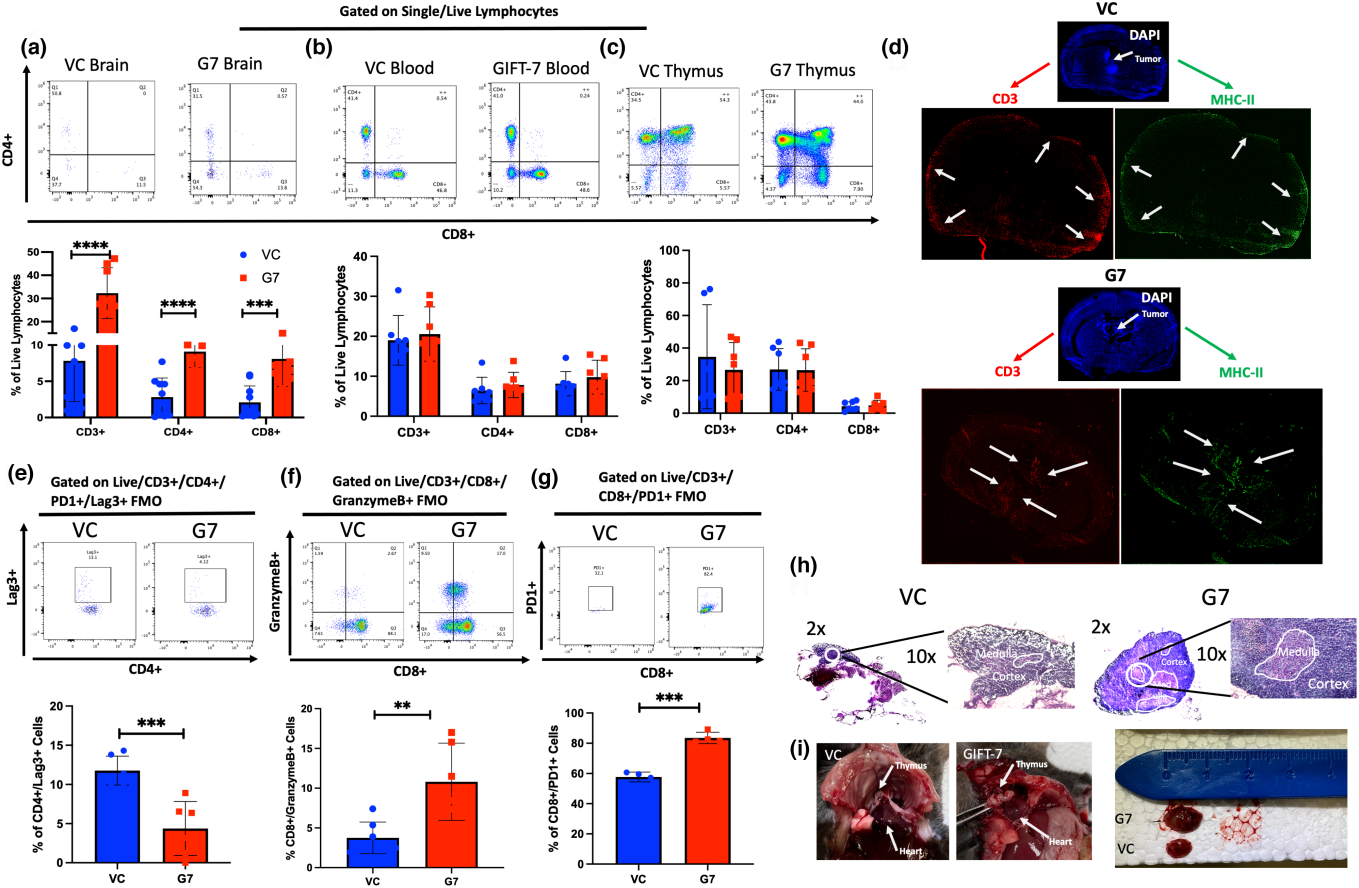
*GIFT‐7 tumor vaccine increases circulating and intratumoral T‐cells*. (a) Gating and quantification for percent of live/CD3+, live/CD3+/CD4+, and live/CD3+/CD8+ T‐cells isolated from the brains of VC and GIFT‐7TVax mice *n* = 9. (b) Gating and quantification for percent of live/CD3+, live/CD3+/CD4+, and live/CD3+/CD8+ T‐cells isolated from the blood of VC and GIFT‐7TVax mice *n* = 9. (c) Gating and quantification for percent of live/CD3+, live/CD3+/CD4+, and live/CD3+/CD8+ T‐cells isolated from the thymus of VC and GIFT‐7TVax mice *n* = 9. (d) Immunofluorescence imaging of sectioned brains of VC and GIFT‐7TVax mice stained with Dapi (blue) anti‐CD3 (red) and anti‐MHCII (green) at 1 week post‐intracranial injection. (e) Gating and quantification of percent of live/CD3+/CD4+/PD1+/Lag3+ cells in VC and GIFT‐7TVax mice *n* = 6. (f) Gating and quantification of percent of live/CD3+/CD8+/GranzymeB+ cells in VC and GIFT‐7TVax mice *n* = 6. (g) Gaiting and quantification of percent of live/CD3+/CD8+/PD1+ cells in VC and GIFT‐7TVax mice *n* = 6. (h) immunohistochemistry images of dissected thymus from VC and GIFT‐7TVax mice 1 week post‐intracranial re‐challenge. (i) Gross examination of VC and GIFT‐7TVax thymus 1 week post‐intracranial tumor re‐challenge. Data in bar graphs are presented as mean +/− SD. Dots in bar graphs (A/B/C/E/F/G) depict individual mice. Statistical significance was determined by corrected ANOVA (A/B/C/E/F/G) **p < 0.01, ***p < 0.001, ****p < 0.0001.

To understand the tumor immune microenvironment and immunologic mechanisms driving complete tumor clearance in the GL261 model, mice were sacrificed 1 week and 3 weeks after intracranial tumor implantation following vaccination, and the immune cell makeup of the blood, brain, and thymus was analyzed. At 1 week post‐intracranial tumor implantation, there was a significant increase in absolute numbers of CD3+, CD4+, and CD8+ lymphocytes within the brain tumor of mice from the GIFT‐7TVax group when compared to VC (VC vs GIFT‐7TVax: CD3+: 7.8% Vs 32.3% *p* < 0.0001; CD4+: 2.8% Vs 9.1% *p* < 0.0001; CD8+:2.1% Vs 8.1% *p* < 0.001) (Figure 2a). This GIFT‐7TVax‐mediated T‐cell increase was unique to the intracranial compartment, as it was not seen within either the peripheral blood or thymic compartments (Blood: VC vs GIFT‐7TVax: CD3+: 19% Vs 20.5% *p* = NS; CD4+: 6.4% Vs 7.8% *p* = NS; CD8+: 8.1% Vs 9.8% *p* = NS; Thymus: VC vs GIFT‐7 CD3+: 34.6% Vs 26.6% *p* = NS; CD4+: 26.8% Vs 26.5% *p* = NS; CD8+: 4.3% Vs 4.8% *p* = NS) (Figure 2b,c). Immunofluorescence (IF) was utilized to visualize the increased intracranial recruitment of T‐cells and demonstrated increased intratumoral and peritumoral CD3 and MHC‐II staining exclusively in the GIFT‐7TVax group 1 week post‐intracranial tumor implantation (Figure 2d). To understand the overall phenotype and functional status of the tumor‐infiltrating T‐cells, exhaustion, cytotoxicity, and overall activation status were profiled using flow cytometry. Intracranial tumor‐infiltrating T‐cells in the GIFT7‐TVax group showed reduced CD4+ T‐cell exhaustion (VC vs GIFT‐7: CD3+/CD4+/PD1+/Lag3+: 11.8% Vs 4.4% *p* < 0.001) (Figure 2e), increased CD8+ cytotoxicity (VC vs GIFT‐7: CD8 + GranB+: 3.7% Vs 10.8% *p* < 0.01) (Figure 2f), and increased activation (Jin et al., 2010) within the CD8+ compartment (VC vs GIFT‐7: CD3+/CD8+/PD1+/Lag3–57.7% Vs 83.6% *p* < 0.001) (Figure 2g). In line with our previous findings (Hsieh et al., 2015) we found a robust increase in the size of the thymus in the GIFT‐7TVax group compared to VC at 1 week post‐intracranial tumor implantation. H&E staining showed an increase in the size of the thymic medullary compartments specifically in the GIFT‐7TVax mice (Figure 2h,i). Further examination revealed that the robust intracranial T‐cell response seen at 1 week is lost by week 3 (Week 3 VC to GIFT‐7: CD8+: 5.0% Vs 1.8% *p* = NS; CD4+: 3.2% Vs 1.2% *p* = NS) (Figure S2a–c). This suggests that the GIFT‐7TVax‐mediated systemic T‐cell response is rapidly mobilized when the tumor is growing, but is subsequently lost once the tumor is cleared from the intracranial compartment (roughly 3 weeks post‐implantation as suggested by Figure 1g). These results demonstrate that GIFT‐7TVax regenerates the aging thymus and increases systemic T‐cell circulation and trafficking to the brain, in response to the tumor.

### 2.3 GIFT‐7TVax results in increased systemic IL‐1β and formation of hyperactive DCs

**FIGURE 3:**
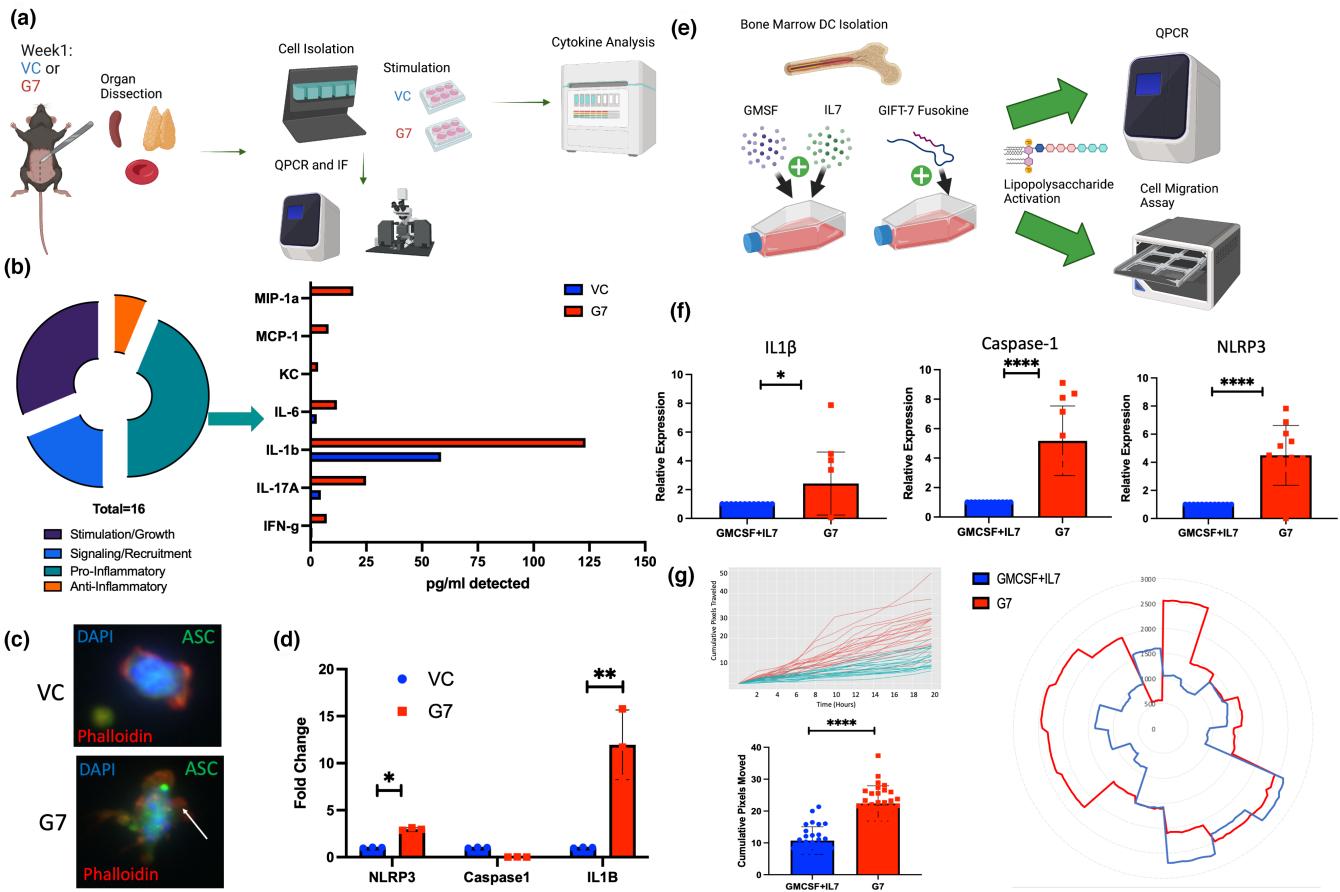
*GIFT‐7TVax results in increased systemic IL‐1B and formation of hyperactive dendritic cells*. (a) Schematic representation of cytokine stimulation, QPCR, and immunofluorescence experiments. (b) Characterization of the broad effects of cytokines contained in the global cytokine screen and pg/ul of detected cytokines from the pro‐inflammatory group. (c) Immunofluorescent staining of isolated spleen dendritic cells from VC and GIFT‐7TVax mice 1 week post‐intracranial challenge. Cells are stained with Dapi (blue), ASC (green), and phalloidin (red). (d) QPCR quantification (fold change) of NLRP3, Caspase‐1, and IL‐1B, in isolated spleen dendritic cells from VC and GIFT‐7TVax mice 1 week post‐intracranial challenge *n* = 3. (e) Schematic of fusokine/cytokine stimulation of BMDCs followed by QPCR and Incucyte movement assay. (f) QPCR quantification (fold change) of IL‐1B, Caspase‐1, and NLRP3, in BMDCs stimulated with either GIFT‐7 (red) or GM‐CSF+IL‐7 (blue) *n* = 12. (g) Line graph, bar chart, and radar plot depicting distance and direction traveled by BMSCs stimulated with GIFT‐7 (red) or GM‐CSF+IL‐7 (blue) *n* = 30 cells. Data in bar graphs are presented as mean +/− SD. Bars in (B) depict average cytokine reading from a biological triplicate and technical duplicate. Dots in bar graphs (D/F/G) depict technical replicates. Statistical significance was determined by corrected ANOVA (D) and Welsh *t*‐test (F/G) *p < 0.05, **p < 0.01, ****p < 0.0001.

T‐cell recruitment to a specific site relies on a complex network of cytokine signaling and secretion (Kohli et al., 2022). To understand global cytokine secretion in the GIFT‐7TVax model, an unbiased cytokine screen was conducted across the blood, thymus, and spleen at 1 week post‐intracranial tumor implantation (Figure 3a). The analysis of pro‐inflammatory cytokines showed a significant increase in IL‐1β in the GIFT‐7TVax mice (Figure 3b, Figure S3a,b). IL‐1β is a pro‐inflammatory cytokine that is produced by several cells within the innate immune system, such as macrophages and DCs (Lopez‐Castejon & Brough, 2011). It is first synthesized in a precursor form and cannot be activated until caspase‐mediated cleavage of the precursor takes place within the inflammasome (Broz & Dixit, 2016; Ghiringhelli et al., 2009; Hanamsagar et al., 2011). Recent studies have demonstrated that increased inflammasome assembly, and thus IL‐1β secretion, can be caused by DC hyperactivation leading to more robust antigen presentation and T‐cell recruitment (Ghiringhelli et al., 2009; Zhivaki et al., 2020). This effect has been shown to boost antitumor response and mediate long‐term immunity in a mouse model of lymphoma and melanoma (Ghiringhelli et al., 2009; Zhivaki et al., 2020). To assay inflammasome formation, we isolated DC's from the spleens of mice 1 week post‐intracranial tumor implantation in both GIFT‐7TVax and VC conditions. Adaptor molecule apoptosis‐associated speck‐like protein containing a CARD (ASC) speck formation staining, a reliable readout of inflammasome formation (Stutz et al., 2013), showed inflammasome speck formation in the DCs from the GIFT‐7TVax mice, while VC mice DCs displayed no speck formation (Figure 3c). For a quantitative measurement, the same cells were subjected to QPCR for the genes present in the inflammasome signaling cascade (Broz & Dixit, 2016). NLRP3 and IL‐1β were significantly increased in the isolated DCs from the GIFT‐7TVax group when compared to VC (VC vs G7: IL‐1β: 12.2‐fold Vs 1‐fold *p* < 0.01; NLRP3: 2.12‐fold Vs 1‐fold *p* < 0.05) (Figure 3d). We also compared DCs isolated from the spleens of unvaccinated mice to VC or GIFT‐7TVax mice which demonstrated no significant difference between the unvaccinated and VC groups (Figure S3c), confirming that GIFT‐7 leads to DC activation. To confirm that the increased inflammasome formation was the result of the GIFT‐7 fusokine mediating DC hyperactivation, we subjected bone‐marrow‐derived DCs (BMDCs) to in vitro stimulation with both recombinant IL‐7 and GM‐CSF (the components of the GIFT‐7 fusokine) and the GIFT‐7 fusokine itself (Figure 3e). Inflammasome cascade markers IL‐1β, Caspase‐1, and NLRP3 were analyzed with QPCR on isolated BMDCs showing robust increases in all genes when stimulated with the GIFT‐7 fusokine compared to its component parts (GM‐CSF+IL‐7 vs GIFT‐7: IL‐1β: 4.5‐fold Vs 1‐fold *p* < 0.05; Caspase‐1: 5.2‐fold Vs 1‐fold *p* < 0.0001; NLRP3: 4.4‐fold Vs 1‐fold *p* < 0.0001) (Figure 3f). Along with increased inflammasome formation, hyperactive DCs are known to be more motile than native DCs (Zhivaki et al., 2020); therefore, we visualized and quantified BMDC movement for 20 hours using an Incucyte incubator insert post‐stimulation with GIFT‐7 or GM‐CSF+IL‐7. DCs stimulated with GIFT‐7 traveled across more pixels and exhibited increased radial movements (quantified by Fiji) compared to GM‐CSF and IL‐7‐stimulated DCs (GIFT‐7 vs GM‐CSF+IL‐7: 22.4 pixels Vs 10.7 pixels *p* < 0.0001) (Figure 3g). To understand whether DC hyperactivation caused by the GIFT‐7 fusokine was a phenomenon in aged rather than young mice, we subjected BMDCs isolated from 6‐ to 8‐week‐old mice to the same QPCR and Incucyte movement tracing as BMDCs isolated from aged mice (above). The results demonstrated no significant enrichment in relevant inflammasome genes between the GM‐CSF + IL‐7 group and the GIFT‐7‐stimulated group (Figure S4d). Furthermore, movement tracing of young BMDCs also demonstrated overall increased movement compared to aged DCs; however, no additional benefit of GIFT‐7 stimulation (Figure S4e). These data indicate that GIFT‐7 exposure results in DC hyperactivation only in aged but not young mice.

### 2.4 GIFT‐7TVax induces RORγt+ Th‐17 lineage effector memory T‐cell and reduces T‐cell exhaustion in LTS mice

**FIGURE 4:**
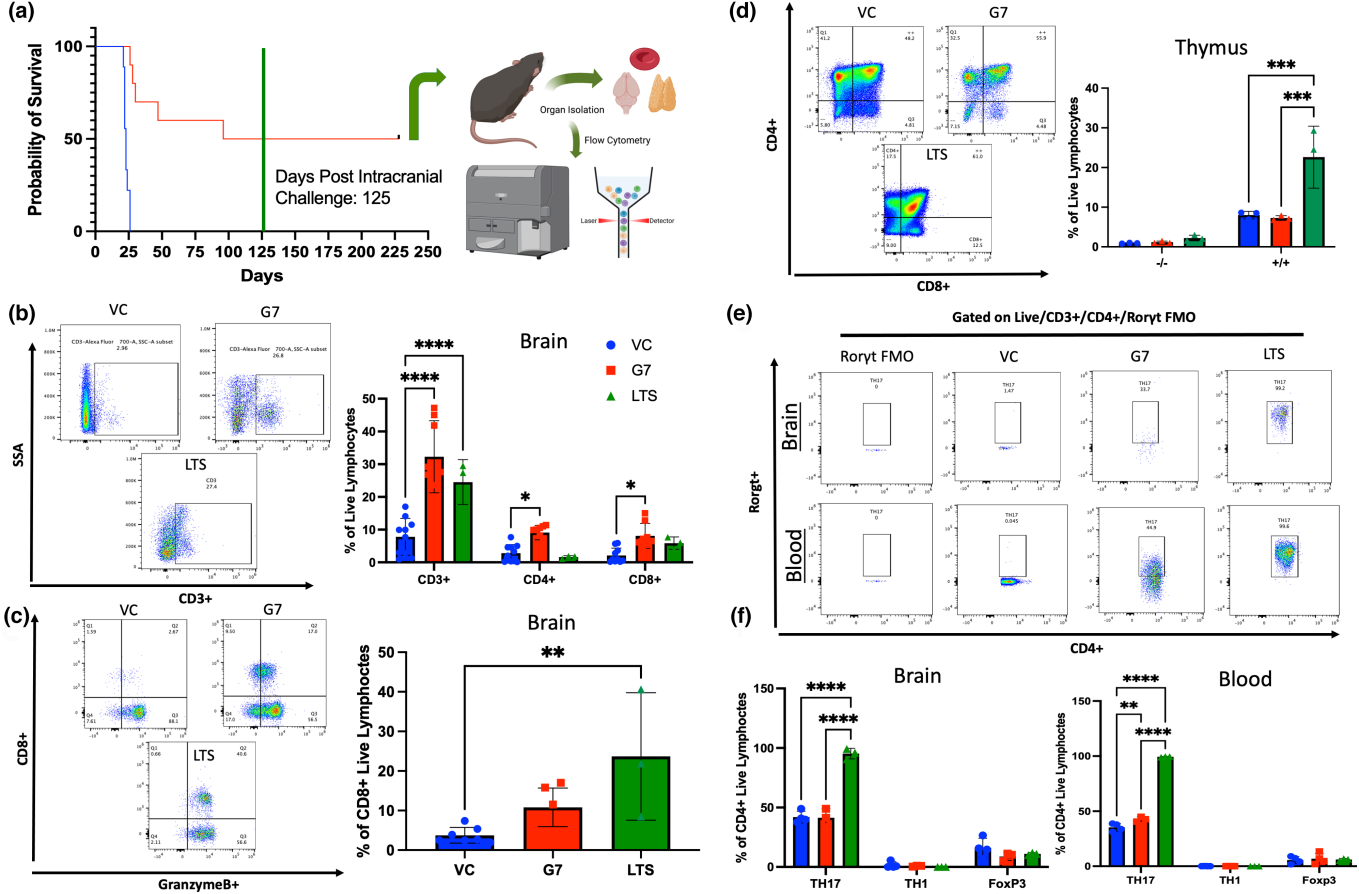
*GIFT‐7TVax induces RORγt + Th‐17 lineage effector memory T‐cells and reduces T‐cell exhaustion in long‐term survivor mice*. (a) Kaplan–Meyer survival graph depicting long‐term survivor mice (day 125 post‐intracranial re‐challenge) and their sacrifice from the experiment for flow cytometry analysis (day 250). (b) Gating and quantification of percent of live/CD3+, live/CD3+/CD4+, and live/CD3+/CD8+, T‐cells in VC, GIFT‐7TVax (1 week post‐intracranial tumor re‐challenge), and long‐term survivor mice (125 days post‐intracranial tumor re‐challenge) *n* = 6 (VC/G7TVax) *n* = 3 (long‐term survivor). (c) Gating and quantification of percent of live/CD3+/CD8+/GranzymeB+ T‐cells in VC, GIFT‐7TVax, and long‐term survivor mice *n* = 6 (VC/G7TVax) *n* = 3 (long‐term survivor). (d) Gating and quantification of percent of live/CD3+/CD4‐/CD8‐, live/CD3+/CD4+/CD8+ T‐cell in VC, GIFT‐7TVax, and long‐term survivor mice *n* = 6 (VC/G7TVax) *n* = 3 (long‐term survivor). (e) Gating strategy for live/CD3+/CD4+/Rorgt+ T‐cells in VC, GIFT‐7TVax, and long‐term survivor mice isolated from the brain and blood including FMO control. (f) Quantification of percent positive live/CD3+/CD4+/Rorgt+, live/CD3+/CD4+/Tbet+, and live/CD3+/CD4+/FoxP3+ T‐cell in VC, GIFT‐7TVax, and long‐term survivor mice isolated form the brain and blood *n* = 6 (VC/G7TVax) *n* = 3 (long‐term survivor). Data in bar graphs are presented as mean +/− SD. Dots in bar graphs (B/C/D/F) depict individual mice. Statistical significance was determined by corrected ANOVA (B/D/F) and Welsh *t*‐test (C) **p < 0.01, ***p < 0.001, ****p < 0.0001.

In the survival experiments, 50% of the mice from the GL261 GIFT‐7TVax group completely cleared their intracranial tumor and became LTS, which was defined as surviving 125 days post‐initial intracranial tumor implantation. At that time point, we re‐challenged the intracranial compartment by again implanting parental tumor cells on the contralateral side and monitored for OS for another 125 days. Interestingly, all of the LTS mice we able to completely reject the re‐implanted tumor on the contralateral side (Figure 4a). To understand the overall immune landscape of the LTS mice, the mice were sacrificed 250 days after initial intracranial tumor implantation and the T‐cell compartment of the blood, brain, and thymus were profiled (Figure 4a). LTS mice had increased levels of CD3+ T‐cell within their brains when compared to VC control mice at 1 week post‐tumor implantation (LTS vs VC CD3+: 24.5% Vs 7.8% *p* < 0.0001). However, they displayed slightly less CD3+, CD4+, and CD8+ T‐cell in the brain compared to GIFT‐7TVax mice 1 week post‐tumor implantation, although a statistically significant difference was not reached (LTS vs GIFT‐7TVax: CD3+: 24.5% Vs 32.3% *p* = NS; CD4+: 9.1% Vs 1.6% *p* = NS; CD8+: 8.1% Vs 5.7% *p* = NS) (Figure 4b). CD8+ T‐cell from LTS mice displayed increased GranzymeB levels when compared to VC mice (LTS vs VC: CD3+/CD8+/GranB+: 23.7% Vs 3.7% *p* < 0.01) (Figure 4c). Furthermore, LTS mice also had significantly increased numbers of CD4+/CD8+ thymic precursors, which have been shown to be the origin of both CD4+ helper and CD8+ cytotoxic T‐cells (Germain, 2002), when compared to both VC and GIFT‐7TVax mice at 1 week post‐intracranial tumor implantation (LTS vs VC: CD4 + CD8+: 22.6% Vs 8% *p* < 0.001; LTS vs GIFT‐7TVax: CD4 + CD8+: 22.6% Vs 7.3% *p* < 0.001) (Figure 4d). CD4+ memory subtypes, specifically Th‐17 T‐cells, can become long‐lived effector memory cells and enhance durable immune response (Kryczek et al., 2011). It is also been observed that Th‐17 cells migrate more effectively to the central nervous system (CNS) parenchyma in autoimmune disorders such as multiple sclerosis, due to their secretion of IL‐17 and IL‐22 which can disrupt the tight junctions of the BBB (Hirota et al., 2007; Okada & Khoury, 2012; Yamazaki et al., 2008). To understand whether a T‐cell lineage compartment shift was responsible for the durable antitumor response that had occurred in the LTS mice, the CD4+ memory compartment was assayed in both the brain and blood using FoxP3 (T‐reg), Tbet (Th‐1), and RORγt (Th‐17) lineage markers. No statistically significant difference was observed between VC, GIFT‐7TVax at 1 week, and LTS mice across the Th‐1 and T‐reg lineages; however, a strong shift toward the Th‐17 lineage was observed, in both the brain and blood of LTS mice, compared to either GIFT‐7TVax at 1 week or VC (Blood: VC vs LTS: Th‐17: 33.4% Vs 99.4% *p* < 0.0001; GIFT‐7TVax vs LTS: Th‐17: 42.7% Vs 99.4% *p* < 0.0001) (Brain: VC vs LTS: Th‐17: 41.8% Vs 95.3% *p* < 0.0001; GIFT‐7TVax vs LTS Th‐17: 41.9% Vs 95.3% *p* < 0.0001) (Figure 4e,f). There was also a strong maintenance of the effector memory subtype in both the CD4+ and CD8+ lineages across the blood and brain of LTS mice (Figure S4a–c). This effector phenotype was further confirmed with cytokine analysis on CD4+/RORγT+ cells from LTS mice. Both IL‐17 and TNFα were detected in the RORγT population present in the blood and brains of LTS mice (Figure S4d). These data indicate that a GIFT‐7TVax‐mediated CD4+ Th‐17 effector memory phenotype promotes intracranial tumor clearance and generates long‐term immunity against glioma.

### 2.5 GIFT‐7TVax remodels the TCR landscape by increasing overall TCR repertoire clonality during early antitumor response

**FIGURE 5:**
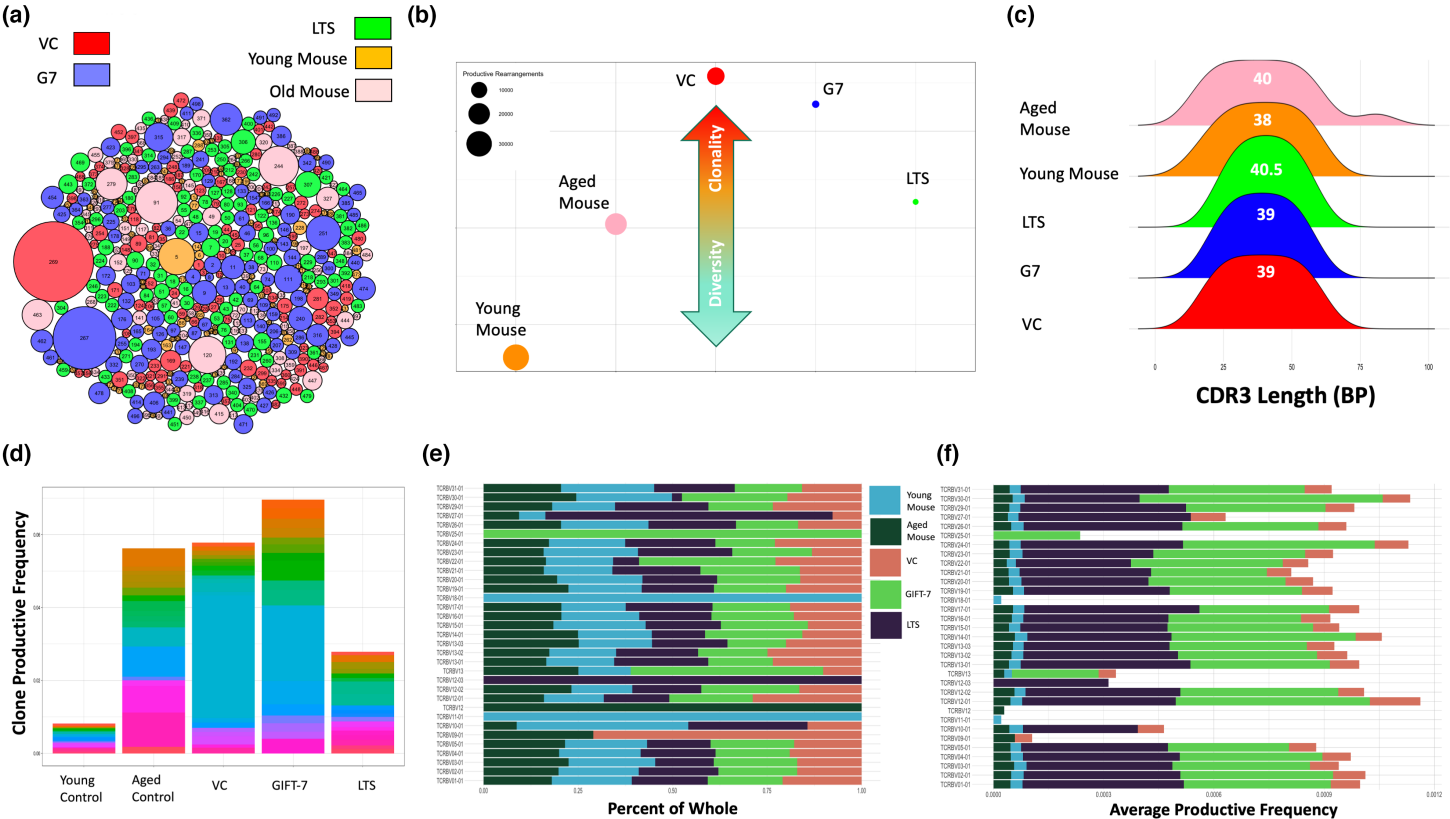
*GIFT‐7 vaccination remodels the TCR landscape by increasing overall TCR repertoire clonality during early antitumor response*: (a) Bubble plot depicting top 100 TCR clones across VC, GIFT‐7TVax, GIFT‐7 long‐term survivor, young mouse (5 weeks), and aged mouse (52 weeks). Size of bubble represents frequency of clone within population, number within bubble represents number of the clone. (b) Plot of Simpson clonality across VC, GIFT‐7TVax, GIFT‐7 long‐term survivor, young mouse, and aged mouse. Higher Simpson clonality indicates a less diverse TCR repertoire while lower Simpson clonality indicates a more diverse TCR repertoire. Size of bubble indicates total productive rearrangements within sample. (c) Ridges plot demonstrating distribution of CDR3 transcript length across VC, GIFT‐7TVax, GIFT‐7 long‐term survivor, young mouse, and aged mouse. Mean CDR3 transcript length is depicted within the ridge bar. (d) Stacked bar chart visualizing the top 20 productive clonal rearrangements across VC, GIFT‐7TVax, GIFT‐7 long‐term survivor, young mouse, and aged mouse. Each hue change within the plot indicates a unique clone. (e) Stacked bar chart indicating TCRB gene usage across VC, GIFT‐7TVax, GIFT‐7 long‐term survivor, young mouse, and aged mouse. Usage is represented as a percent of the whole TCRB gene utilization across samples. (f) Stacked bar chart indicating TCRB gene usage across VC, GIFT‐7TVax, GIFT‐7 long‐term survivor, young mouse, and aged mouse. Bars represent average productive frequency of the TCRB gene across samples. Data are presented as individual clones (A/D), sample productive rearrangements (B), and individual TCRB genes (E/F).

Since GIFT‐7TVax regenerated the thymus, the primary organ for T‐cell formation and antigen education (Alves et al., 2009), and increased intracranial T‐cell presence was only seen in the GIFT‐7TVax group, we hypothesized that GIFT‐7 preferentially generated/expanded tumor‐associated T‐cell. To test this hypothesis, we analyzed the TCR landscape of the GIFT‐7TVax mice during initial antitumor immune response, at 1 week post‐intracranial tumor implantation, as well as LTS mice with durable antitumor immunity, and compared them with young, aged, and VC mice. We performed bulk TCR sequencing using isolated genomic DNA from peripheral blood. Several different measures, including rearrangement productive frequency, CRD3 length, clonal commonality, and TCRβ gene usage, were utilized across the groups to analyze repertoire diversity and composition. Global visualization of repertoire composition indicated expansion of dominant clones and repertoire focusing within the tumor‐bearing mice both the VC and GIFT‐7TVax groups at the onset of the antitumor immune response, demonstrated by clone #267 (GIFT‐7TVax) and #269 (VC) (Figure 5a). LTS mice, which represent a longer‐term durable immunity against tumor, displayed less repertoire focusing and less dominating clones, similar to both the young and aged non‐tumor bearing mice (Figure 5a). Simpson clonality, which approaches a value of 1 if a repertoire is entirely monoclonal and a value of zero if a repertoire is completely unique (Qi et al., 2014; Rosati et al., 2017), was utilized to understand the composition of the T‐cell repertoires among our analyzed groups. In general, the repertoires within the groups were quite diverse and showed little similarity to one another when compared with Morisita Index (Figure S5a,b). A significant increase in the clonality of repertoires was observed in the tumor vaccinated groups (VC or GIFT‐7TVax) when compared to the young non‐tumor‐bearing mice (Dunn multiple comparison correction: VC to Young Control: 0.024 Vs 0.006 *p* < 0.05; GIFT‐7TVax to young control: 0.018 Vs 0.006 *p* < 0.05) (Figure 5b). In addition, GIFT‐7TVax at 1 week and LTS mice specifically showed a large decrease in the number of total productive β‐rearrangements present indicating a possible shift away from the αβ phenotype and toward the γδ phenotype (Burtrum et al., 1996; Joachims et al., 2006) (Figure 5b). γδ T‐cell have been shown to support the development of CD4+ Th‐17 cells, which are enriched in the LTS mice (Figure 4f), through the secretion of specific cytokines such as RANTES, MCP‐1, IL‐17, and IP‐10 (Korn et al., 2009; Murzenok et al., 2002; Paul & Lal, 2016; Pistoia et al., 2018). Global cytokine analysis for γδ and Th‐17‐related cytokines showed increase in each of the above cytokines in GIFT‐7TVax mice compared to VC (VC Vs GIFT‐7TVax RANTES:11.09 Vs 67.15; MCP‐1: 0 Vs 8.07; IL‐17: 4.60 Vs 24.91; IP‐10: 20.33 Vs 125.14) (Figure S5c). There was little difference in the mean CDR3 transcript length between each of our groups, and while the aged non‐tumor‐bearing mice did exhibit larger standard deviation in CDR3 transcript lengths relative to the other groups, statistical significance was not reached (standard deviation age mouse: 17.39 vs young mouse: 13.76, LTS: 10.81, VC:13.42, G7: 11.68; *F*‐test = NS) (Figure 5c). Clone productive frequency for the top 20 clones was similar across the aged control, VC, and GIFT‐7TVax mice, and significantly lower in the young control mice while moderately lower in the LTS mice (GIFT‐7TVax Vs Young Mouse: 0.003 Vs 0.0004 *p* < 0.05, G7 vs LTS 0.003 Vs 0.002 *p* = NS) (Figure 5d). Finally, TCRβ gene usage remained roughly proportionally consistent between groups (Figure 5e); however, when productive frequency of the gene usage was measured, both GIFT‐7Tvax at 1 week and LTS mice showed higher overall TCRβ gene usage (VC Vs GIFT‐7TVax:0.0007 Vs 0.003 *p* < 0.00001; VC Vs LTS: 0.0007 Vs 0.004 *p* < 0.0001; G7 Vs LTS 0.003 Vs 0.004 *p* = NS) (Figure 5f). Interestingly, unique TCRβ gene usage was displayed in both the GIFT‐7TVax group (TCRBV25‐01) as well the LTS group (TCRBV12‐03), indicating a likely tumor‐related clonal expansion. Individual clone tracking between groups also indicated little overall similarity with the highest frequency of shared clones occurring between the non‐tumor‐bearing young and aged mice (Morisita Index 0.004, similar clones: 115) (Figure S5d–f). Aging itself has been shown to have a significant impact on the TCR repertoire and in concordance with other groups studying age effects on the TCR repertoire (Ahmed et al., 2009; Britanova et al., 2014; Naylor et al., 2005; Vallejo, 2006), we found a difference in T‐cell repertoire makeup between the young and aged non‐tumor‐bearing mice. Young mice exhibited more repertoire diversity and less total clone productive frequency per rearrangement (Figure 5b,d), as well as a differential usage of TCRB genes both proportionally and in productive frequency (Figure 5e,f). These data show the clonal focusing of the TCR repertoires of vaccinated mice which relaxes somewhat as the tumor is cleared and homeostasis is re‐achieved, retaining a possible γδ mediated durable Th‐17 memory for long‐term tumor‐specific immunological memory. Furthermore, the stark difference between the repertoires of aged and young mice independent of any experimentation is clear and most likely plays a role in the more robust antitumor response on vaccination seen in the young mice (Figure S1d).

### 2.6 GIFT‐7TVax is therapeutic and increases OS in a clinically relevant aged mice model of glioma

**FIGURE 6:**
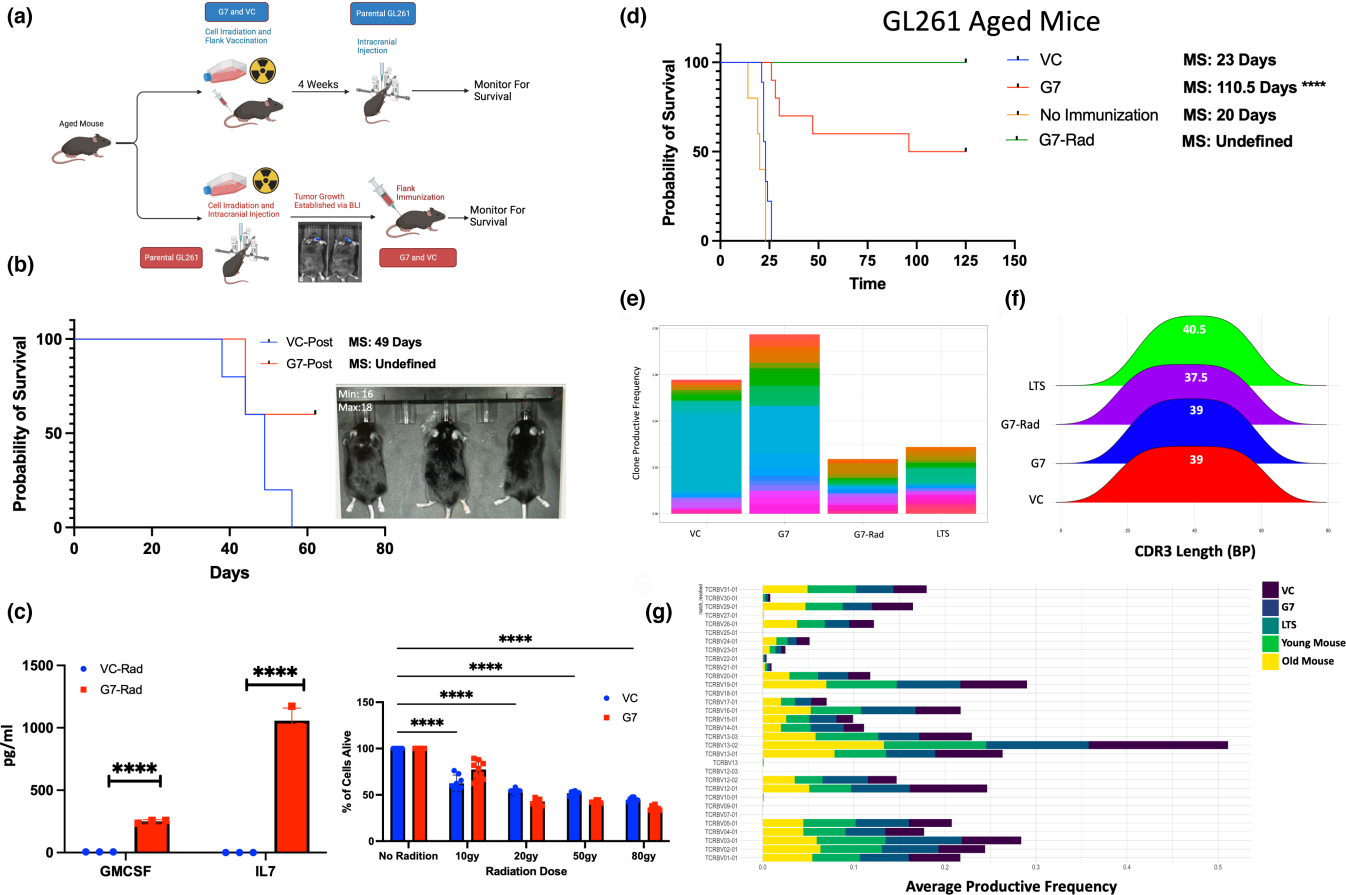
*GIFT‐7TVax is therapeutic and increases overall survival in a clinically relevant aged mice model of glioma* (a) schematic describing in vivo post‐intracranial implantation GIFT‐7TVax as well as G7‐Rad. (b) Kaplan–Meyer curve depicting survival for VC immunization post‐intracranial injection (purple) and GIFT‐7Tvax post‐intracranial injection (orange). (c) Quantification of cytokine secretion (IL‐7 and GM‐CSF) post 50gy radiation treatment in VC and GIFT‐7 transfected GL261 tumor cells *n* = 3 (left) and GL261 transfected VC and GIFT‐7 cell survival post 10, 20, 50 and 80Gy of radiation *n* = 6 (right). (d) Kaplan–Meyer survival curve depicting survival for no immunization (yellow), VC immunization (blue), GIFT‐7TVax (red), and G7‐Rad (green). (e) Stacked bar chart indicating productive frequency of the top 20 clones across VC, GIFT‐7TVax, G7‐Rad, and GIFT‐7 long‐term survivor. Each hue within the plot and group depicts a separate clone. (f) Ridge plot depicting the mean CDR3 transcript length across VC, GIFT‐7TVax, G7_Rad, and GIFT‐7 long‐term survivor. Mean CDR3 transcript length is displayed within the ridge. (g) Stacked bar chart indicating the average productive frequency of detected TCRB genes within VC, GIFT‐7TVax, G7‐Rad, and GIFT‐7 long‐term survivor. Bars display the average productive frequency of the TCRB gene utilized across the samples. Data are presented as mean +/− SD (C), individual clones (E), and individual TCRB genes (G). Statistical significance was determined by Welsh *t*‐Test (C left) and corrected ANOVA (C right). ****p < 0.0001.

In the clinical setting, tumor vaccines can only be administered after tumor is diagnosed and treated with the initial standard of care. GBM IDHwt standard of care consists of surgical resection, followed by radiation and concurrent chemotherapy, along tumor‐treating fields and adjuvant chemotherapy to manage residual disease (Stupp et al., 2005, 2017). To replicate the clinical treatment paradigm, we explored a clinically relevant scenario where a standard GL261 tumor, which was irradiated (50gy) to simulate the clinical standard of care in GBM IDHwt, was implanted and allowed to establish tumor (visualized by bioluminescence imaging (BLI)). After tumor establishment was confirmed with BLI, mice were then peripherally vaccinated with GIFT‐7TVax or VC and monitored for OS (Figure 6a). We found that even in the post‐tumor implantation vaccination model, the group vaccinated with GIFT‐7TVax has significantly better OS compared to the VC (OS: GIFT‐7TVax Vs VC 49 days Vs undefined) with survivors demonstrating complete tumor clearance via BLI (Figure 6b). To make the tumor vaccination model translatable to human clinical trials, such as the previously trialed GM‐CSF tumor vaccine GVAX (Ho et al., 2021; Le et al., 2015; Nair et al., 2020; Wu et al., 2020), we also generated an irradiated GIFT‐7TVax. VC or GIFT‐7 transfected tumor cells were radiated (50Gy) prior to flank vaccination. To ensure radiation did not affect the fusokine production or viability of the GIFT‐7TVax cells, we performed a cell killing assay as well as ELISA post‐radiation. ELISA demonstrated continued IL‐7 and GM‐CSF secretion by the irradiated GIFT‐7TVax cells compared to irradiated VC cells (GM‐CSF: VC Vs GIFT‐7: 3.05 pg/mL–354.5 pg/mL *p* < 0.0001; IL‐7: VC Vs GIFT‐7: 0.0 pg/mL–1716 pg/mL *p* < 0.0001) (Figure 6c). The cell viability assay confirmed the presence of viable cells in both VC and GIFT‐7 group post‐radiation (No Rad vs Rad: VC 100%–50.1% live *p* < 0.0001; G7: 100%–40.5% live *p* < 0.0001) (Figure 6c). The mice were peripherally vaccinated similar to our initial experiment with irradiated GIFT‐7TVax and monitored for survival. Interestedly, irradiated GIFT‐7TVax resulted in complete intracranial tumor clearance in 100% of the mice compared to 50% LTS in the non‐radiated GIFT‐7TVax (MS no vax: 20 days, VC: 23 days, G7: 110.5 days, radG7: undefined) (Figure 6d). TCR sequencing analysis was also conducted on mice who received irradiated GIFT‐7TVax to determine the impact of the radiation on the T‐cell repertoire. Productive frequency across the top 20 clones of each group showed a large reduction compared to both VC and GIFT‐7Tvax, with similar productive frequency as the LTS mice from non‐irradiated group (Figure 6e). CDR3 transcript length remained similar between all the groups (Figure 6f) as did productive TCRβ gene usage (Figure 6g). Clone overlap between groups was also minimal as measured by Morisita Index as well as raw overlap (Figure S6a–c). Finally, raw nucleotide overlap between the four groups also indicated low similarity with only one sequence being represented in every group (Figure S6d). Interestingly, the irradiated GIFT‐7TVax did result in significantly more unique nucleotides than either GIFT‐7TVax or LTS mice (Figure S6d). To fully establish the clinical translation potential of this treatment modality, we transfected GBM IDHwt cells isolated from fresh tumor samples from patients after surgical resection (obtained from UW Hospital) and validated their ability to synthesize and secrete the GIFT‐7 fusokine (P95b: VC Vs G7: 0 pg/mL Vs 590 pg/uL *p* < 0.0001; P96b: 7 pg/uL Vs 587 pg/uL *p* < 0.0001) (Figure S6e). These results demonstrate the relevance and efficacy of GIFT‐7TVax vaccination in clinically applicable therapeutic scenarios, establishing the foundation for translation into a phase I clinical trials.

## 3 DISCUSSION

Increasingly, age‐related immune senescence is being recognized as an independent negative prognostic (Aronow, 2006; Berger et al., 2005; Effros et al., 2005; Falci et al., 2013; Ladomersky et al., 2020; Lamb et al., 2016; Murman, 2015; Muthukumar et al., 2004; Nair et al., 2020; White et al., 2014) factor in many disease processes; however, treatments tailored specifically to an aged population are rare. In the context of GBM IDHwt, nearly all of the current clinical immunotherapies, DC vaccination, immune‐checkpoint blockade, CAR‐T‐cell therapy, require a well‐functioning immunological baseline to generate effective and durable adaptive antitumor immune response (Mogilenko et al., 2022; Vallejo, 2006; Weiskopf et al., 2009). It has been demonstrated repeatedly that there is dramatically reduced vaccine response and vaccine longevity in elderly due to alerted adaptive immune response (Boraschi et al., 2013; Effros, 2004; Naylor et al., 2005; Ongrádi et al., 2009; Vallejo, 2006; Weiskopf et al., 2009). Furthermore, we and others have shown that by driving immune senescence GBM IDHwt may itself accelerate age‐related immune dysfunction (Falci et al., 2013; Huff et al., 2019, 2021; Kanesvaran et al., 2018; Ladomersky et al., 2020; Yousefzadeh et al., 2021). The culmination of these processes results in the most successful responders to GBM IDHwt therapies, conventional or novel, being young individuals (Tykocki & Eltayeb, 2018).

A fundamental observation made in this study was that a peripheral vaccination strategy works differently in young vs aged hosts. Young mice given a peripheral vaccination without GIFT‐7 were able to successfully clear intracranial tumor; however, aged mice demonstrated no peripheral vaccination effect without GIFT‐7. Currently, the majority of glioma pre‐clinical mouse work is done on 6‐ to 8‐week‐old mice, which does not capture the effect of aging immune system seen in the GBM IDHwt patient population. Because of this, the age of the host should be taken into account when designing experiments, especially ones that rely on the baseline robustness of the immune system. Since we started all our experiments in mice aged 52 weeks, many of our LTS mice experiments were done on mice over 80 weeks old and even in this population GIFT‐7TVax generated lasting immunity.

**FIGURE 7:**
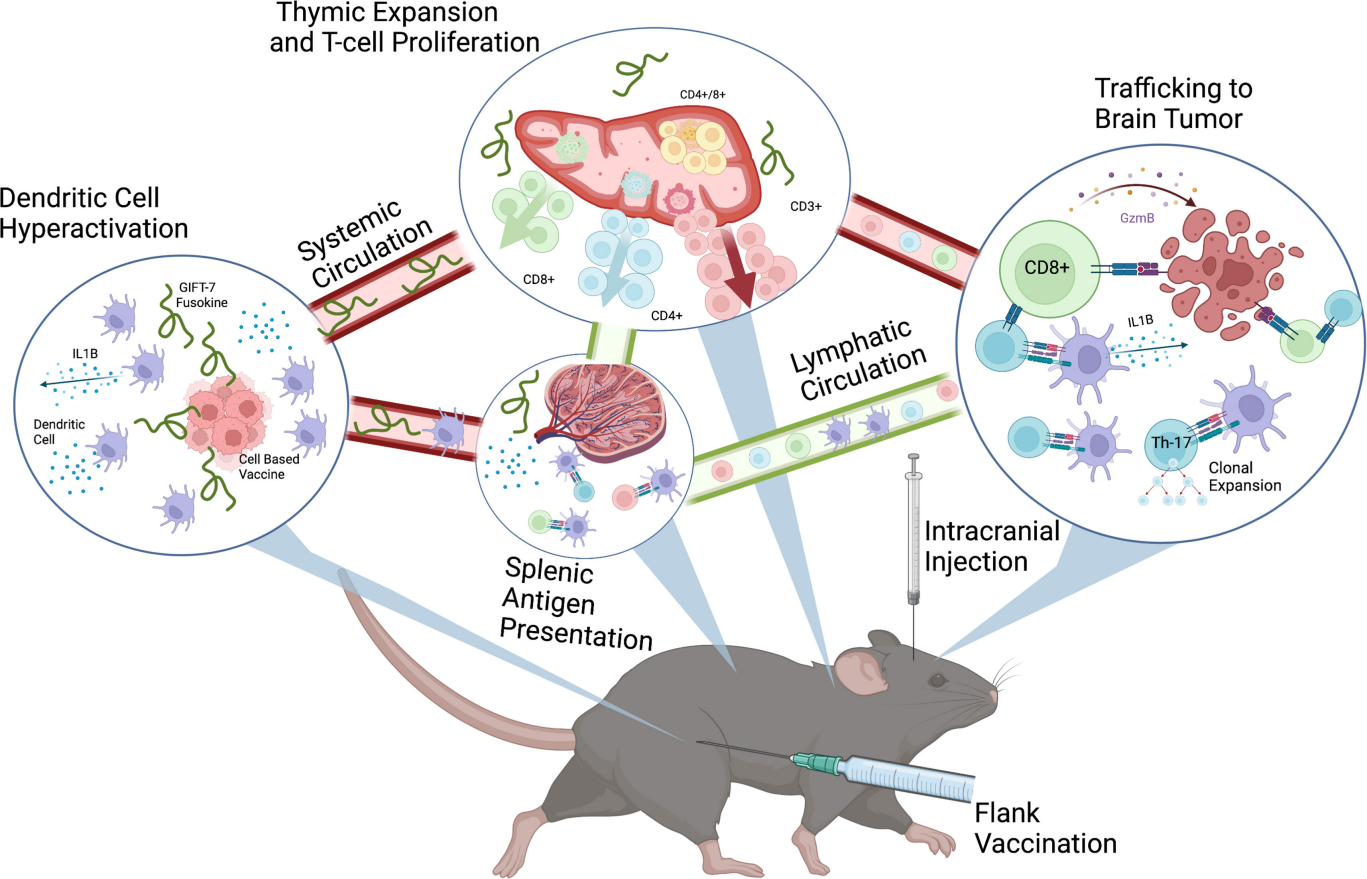
Mechanistic overview of GIFT‐7TVax effects.

Our data demonstrate an effective and durable tumor vaccination strategy, mediated by the fusokine GIFT‐7, against a fatally incurable cancer uniquely in an aged model (Gómez‐Oliva et al., 2021; Haddad et al., 2021; Jacobs et al., 2011) (Figure 7). We found that GIFT‐7TVax is efficacious in multiple glioma rodent models in prolonging survival but is durable in the more immunogenic GL261 model. GL261 is a widely used (Gómez‐Oliva et al., 2021; Haddad et al., 2021; Jacobs et al., 2011) and accepted syngeneic mouse model of glioma and is ideally suited for pre‐clinical proof of concept studies of antitumor T‐cell response due to its higher mutational burden, compared to human GBM IDHwt (Dunn et al., 2020; Johanns & Dunn, 2017). In this model, we found that GIFT‐7TVax regenerated the aged thymus resulting in increased T‐cell recruitment and trafficking to the brain at the onset of the antitumor immune response. More importantly, it produced LTS that displayed durable Th‐17 long‐term immunity in half of the mice treated. These LTS mice subsequently rejected contralateral tumor re‐challenge 100% of the time. To make this vaccination strategy clinically applicable, we created an irradiated GIFT‐7TVax that continues to produce GIFT‐7 with limited cell proliferation capabilities. This strategy of creating irradiated live tumor vaccine has been tested and proven safe in clinical trials (Ho et al., 2021; Le et al., 2015; Nair et al., 2020; Wu et al., 2020). Remarkably, vaccination with irradiated GIFT‐7TVax produces durable long‐term immunity in 100% of the treated animals, likely due to increased immunogenicity of the irradiated tumor vaccine, which was evident from several unique TCR rearrangements seen in this group. In addition, we demonstrated that the GIFT‐7TVax strategy is also efficacious as a therapeutic vaccine when adapted to a more relevant clinical scenario in which mice with pre‐established and treated tumors were vaccinated and monitored for survival.

Although the GIFT‐7 fusokine was intended to augment the aged immune system, and its administration results in thymic regeneration and increased T‐cell numbers (Hsieh et al., 2015; Williams & Galipeau, 2011), TCR repertoire analysis show that this T‐cell augmentation is restricted in diversity and geared toward higher clonality likely due to tumor‐specific antigens from the cellular vaccination. This was well established by the sharp contrast in diversity and productivity of the GIFT‐7TVax mice when compared to young non‐tumor‐bearing mice. Indeed, in young mice, we saw no benefit of GIFT‐7TVax outside of the benefit of the VC peripheral tumor vaccination itself, further solidifying that glioma takes advantage of the limited functionality of the aged immune system. In human clinical studies, nivolumab administration was demonstrated to also focus the TCR and result in an increase in CDR3 length among patients, similar to our results in LTS mice (Schalper et al., 2019). An increase in overall TCRα and β clones was also detected within the nivolumab group in contrast to our findings, highlighting likely differences in mouse vs human TCR dynamics (Casrouge et al., 2000; Glusman et al., 2001).

Since GIFT‐7 combines IL‐7, which primarily influences T‐cell compartment, and GM‐CSF, which influences DCs, we interrogated both the DC and T‐cell compartments and found that GIFT‐7 but not its individual component cytokines leads to the formation of hyperactive DCs. Hyperactive DCs are known to be more efficient at migration and antigen presentation and generate long‐lasting durable antitumor immunity (Ghiringhelli et al., 2009; Zhivaki et al., 2020). In the setting of systemic tumor, hyperactive DCs are known to polarize antitumor immune response toward Th‐1 response (Zhivaki et al., 2022); however, in our intracranial tumor model, we found Th‐17 polarized long‐lasting durable memory T‐cells. This represents a possible division between the immunity generated against cancers that are accessible to the peripheral immune system versus cancers that are accessible only to the immune system patrolling the CNS. In fact, recent research has begun solidifying both the complexity and the uniqueness of the CNS immune system (Louveau et al., 2018; Mazzitelli et al., 2022; Møllgård et al., 2023; Rustenhoven et al., 2021), so a difference in their responses to cancer or invasion in general is quite likely. In exploring this effect further, our TCR repertoire analysis of the productive V (D) J rearrangements in our LTS mice suggests a γδ‐mediated Th‐17‐specific effector phenotype induction could be driving this phenomenon. This population may be ultimately responsible for the long‐term immunity conferred within the LTS group. Critically, both γδ T‐cells and Th‐17 effector cells are understudied in the current immunotherapy landscape, which is biased toward αβ CD8+ cytotoxic T‐cell. For γδ T‐cell specifically, new research is highlighting their possible contribution to the antitumor response across cancer types (Kobayashi et al., 2001; Zhao et al., 2018). γδ T‐cells uniquely are not restricted to respond to antigens solely presented by MHC peptides like their αβ counterparts, providing remarkable plasticity and diversity in their immune response (Lafont et al., 2014). Similarly, Th‐17 cells are now being shown to underlie a robust antitumor response in both humans and mice; however, targeted therapies utilizing this lineage are still lacking (Guéry & Hugues, 2015; Kryczek et al., 2011; Punt et al., 2015). Currently, these studies are limited by lack of technology and currently available assays, especially within the mouse model system, we plan to move this forward in future studies by translating this therapeutic strategy in patients and leveraging more advanced sequencing technologies that are available in humans. In fact, an ongoing phase I clinical trial “Novel Gamma‐Delta (γδ) Cell Therapy for Treatment of Patients with Newly Diagnosed Glioblastoma” is currently recruiting patients (NCT04165941). In this trial, γδ T‐cell are gene edited to make them resistant to the cytotoxic effects of temozolomide chemotherapy which may prolong their antitumor effect. This along with further study in humans of the precise mechanisms of γδ T‐cell antitumor effect will be critical in understanding this potentially important but understudied lymphocyte population.

In summary, our study demonstrates the feasibility of targeting a therapy toward the aged immune system and highlights that accounting for age in our animal models of disease is critical. Our GIFT‐7TVax model was able to generate lasting antitumor immunity while still remaining effective when translated to more clinically relevant scenarios, enabling rapid translation of this research to phase I clinical trials. Our results solidify that any therapy that hopes to provide lasting clinical benefit to GBM IDHwt patients will need to both account for the immunological aging present in the disease population, as well as explore novel immunotherapeutic avenues.

## 4 METHODS AND MATERIALS

### 4.1 Animal housing and survival experiments

All animals used in experiments were maintained in accordance with the IRB and IACUC committee rules established at the University of Wisconsin. C57/Black 6 mice used for research were obtained from Charles River laboratories. Ages of the mice used in the experiments are noted specifically within the results section and ranged from 6 to 87 weeks. All mice were contained in a temperature‐controlled animal room maintaining a 12‐h light and dark cycle.

For tumor implantation and survival experiments cells were removed from culture plastic with trypsin and confirmed to be in single‐cell suspension. The suspension mixture was then counted using a Biorad cell counter with Trypan blue dye used to exclude dead cells. Once the desired count of cells was obtained, a solution containing phosphate‐buffered saline along with the appropriate number of cells was created. Under sterile conditions in a biosafety hood, mice were anesthetized using ketamine, the scalp was shaved and cleaned, and an incision and burr hole were made to the skull. A maximum of 5μl of cell suspension was injected into the brains of the mice using a stereotactic frame with coordinates 2 mm behind the bregma suture and 1.5 mm posterior to the midline suture. The skin incision was closed using sterile vicryl sutures and mice were given post‐procedure analgesics (buprenorphine) to ease discomfort. In all animal experiments, a straight 50/50 breakdown of males and females was maintained.

For flank vaccination, cells were obtained as described above and injected into the right flank of the desired mouse using a sterile insulin syringe with no injection volume exceeding 150μl. Flank tumors were monitored throughout the life of the mouse and were not allowed to exceed veterinary defined sizes or become ulcerated/necrotic. Flank tumor was measured at indicated times using calipers and recorded and plotted using Graphpad Prism.

### 4.2 Cell culture and DC isolation

All cells utilized for experimentation were maintained in a sterile culture within a Thermo Fischer Vios cell incubator at 37 degrees C with 5% CO_2_ supplementation. Mouse tumor lines GL261 and CT2A were maintained with DMEM supplemented with 10% fetal bovine serum and 5% penicillin/streptomycin antibiotic. DCs were maintained in RPMI supplemented with IL‐4 (10 ng/mL), GM‐CSF (10 ng/mL), and Glutamax (100×) growth factors as well as b‐mercaptoethanol (1 ng/mL) and penicillin/streptomycin antibiotic (5%).

DCs were isolated from young as well as aged mouse bone marrow progenitor cells as per previously published protocol (Matheu et al., 2008). Briefly, mouse femurs were dissected, and muscle removed under sterile conditions, a break in the femur was induced on each side and marrow flushed out using blank RPMI through an insulin syringe. Marrow was then broken up via pipetting to create a single‐cell suspension which was then plated on non‐adherent tissue culture dishes utilizing the above media recipe. DCs were allowed to form for progenitors for 7 days before use in experiments.

### 4.3 Immunohistochemistry and immunofluorescence

Immunohistochemistry was carried out on 8‐micron sections of frozen brains after an overnight soak in a sucrose solution. Sections were mounted to cover‐glass and stained with hematoxylin and eosin and then sealed for brightfield imaging on a Keyence microscope. For immunofluorescence on mouse brain sections, the sections were blocked using goat serum and then stained in with surface antibodies for MHC‐II and CD3 overnight (antibodies used included in supplementary documentation). After overnight staining, the antibodies were washed away and the sections were sealed for visualization on a Keyence fluorescence microscope.

### 4.4 Fusokine transfection

Lentiviral constructs for fusokine expression were obtained from our collaborators (Hsieh et al., 2015) and transfected into both GL261 and CT2A using a lentiviral vector system and lipofectamine transfection reagent. Puromycin selection and flow cytometric sorting were utilized to purify a transfected population. For fusokine transfection in human GBM IDHwt patient samples, a similar method was utilized without the inclusion of the puromycin selection due to observed cell line toxicity. Successful transfection was confirmed by both fluorescence staining (GFP tag contained in lentiviral construct) and ELISA for component cytokines (GM‐CSF/IL‐7). ELISA kits were purchased from Thermo and utilized according to the manufacturer's instruction and readout was visualized on Molecular Devices fluorescent plate reader.

### 4.5 Inflammasome formation assays

For stimulation with fusokine or cytokines, DC media was removed and blank RMPI containing the necessary cytokine was added. Fusokine stimulation on DCs was carried out as described in (Hsieh et al., 2015). DCs were incubated with fusokine/cytokine for 3 h and then activated with lipopolysaccharide (1 μg/mL) overnight. Before collection for experiments, DCs were also primed with ATP (1 μg/mL) 10 min prior to collection. Once collected, activated DCs were lysed and RNA was collected (Quiagen RNEasy) for QPCR as per standard protocols (Applied Biosystems Instrumentation). Primers were designed using Primer Blast (NCBI) and de‐novo synthesized by Thermo‐Fischer. Sequences for primers are included in supplemental documentation.

For IF inflammasome visualization, cytokine/fusokine stimulation was carried out as above. Cells were then fixed (PFA), blocked (Goat Serum), and stained with anti‐ASC (find), Phalloidin (Sigma), and Dapi mounting media (Fischer). Slides were visualized on a Keyence fluorescence microscope.

### 4.6 DC movement assay

Dendric cell movement post‐cytokine/fusokine stimulation was visualized using an Incucyte (Sartorius) incubator within a standard cell culture incubator. Fusokine/cytokine stimulation was carried out as described above. Stimulated and activated DCs were serially photographed every 2 h for 20 h and the resulting image files were stitched together using Fiji. Manual cellular movement tracing was conducted in Fiji for *n* > 30 cells in each condition. Traces from two independent individuals were averaged to create a resulting data file that was plotted in both R and Excel. Radar charts were generated in excel using data from >30 DC traces compiled in Fiji and normalized to start at a common center point.

### 4.7 Flow cytometry

Flow cytometry was carried out as described in (Huff et al., 2019, 2021). Briefly, mice were sacrificed, and the relevant organs were dissected out and filtered through 70 micro mesh creating a single‐cell solution. The lymphocyte population was enriched in brain samples using a Percoll density centrifugation gradient. Blood lymphocytes were enriched utilizing an ACK lysis buffer treatment to remove red blood cells. Cellular staining was carried out according to the antibody manufacturer's instructions (antibody list included in supplemental materials). For cytokine assay, stimulation was conducted on cells using the Cell Stimulation Cocktail with Protein Transport Inhibitor (Thermo) diluted to 1× from 500× and supplemented into the media of isolated cells which were left in an incubator for 6 h prior to antibody staining. Design of flow cytometry staining panels was checked for fluorophore overlap using Thermo's panel design tool. Flow cytometry was carried out an Attune flow cytometers (Thermo) with multi‐antibody compensation as well as FMO controls. Analysis gates were set based on live lymphocytes (Fixable Yellow Viability Dye Thermo) and then relevant FMO control. Flow analysis was conducted in Flowjo with results plotted using Graphpad Prism.

### 4.8 Global cytokine screening

Global cytokine screening was conducted using Isoplexis Spark instrumentation along with Isoplexis Mouse Adaptive Immune assay chip according to the published manufacturer's protocol (https://isoplexis.com/support/sample‐preparation/). Briefly, organs were isolated from sacrificed mice in the various treatment groups and filtered through 70‐micron mesh to create single‐cell solutions. Cells were plated in triplicate in 96‐well dishes and subject to PMA/Ionomycin stimulation for 24 h prior to cytokine analysis. Pooled conditioned media from isolated triplicates was loaded onto the Isoplexis mouse adaptive immune chip which was then run on the Isoplexis spark. Cytokine‐level analysis was checked against background levels using Isoplexis quality control metrics on the Isospark machine. Result measurements from the analysis represent a biological triplicate of conditioned media per organ analyzed as well as an average of a technical duplicate run on the chip itself. All reported effects were marked as significant between groups and above background detection by the Isospark instrument. Results were pulled from the Isospark software and plotted using Graphpad.

### 4.9 TCR sequencing

TCR sequencing was carried out by Adaptive Biotechnologies according to standard company protocols. Experimentally, mice from each group were sacrificed and blood draw was obtained through cardiac puncture. Isolation of genomic DNA was performed in the laboratory using a Quiagen DNEasy Blood and Tissue Kit and gDNA quality and concentration was confirmed using a Nano‐Drop fluorimeter (Thermo). Isolated gDNA was then sent to Adaptive headquarters where proprietary primer sets were utilized to isolate and amplify the TCRB gene locus within in mouse genome. Sequencing of the resulting cDNA libraries was performed at Adaptive's facility and PCR duplication bias was eliminated using established company quality controls. Both raw and quantified data were transferred back to the laboratory using Adaptive's data analysis cloud. Raw data were analyzed using MiXCR (Bolotin et al., 2015) while quantified data were mined and plotting using R (version 4.1).

### 4.10 Cellular radiation

Radiation of cells was done in a contained and biologically shielded gamma radiation generating instrument within the Small Animal Radiation and Imaging Facility at UW‐Hospitals. Cells were radiated well adhered to their culture dishes and given 24 h to recover prior to standard tissue culture collection for use in experiments. Doses of radiation for cells lines were calculated by the radiation machine software according to size and shape of culture dishes. The same instrument and radiation dosing protocol were used throughout the entire study to minimize variation in radiation doses received by cells. In vivo injection of radiated cells was carried out as described in the animal use section.

### 4.11 Statistical analysis

Statistical analysis for the project was conducted using both R (version 4.1) and Graphpad Prism. Biorender (Biorender.com) was used for schematic diagram and graphical abstract creation. Where bar graphs are used individual data points are shown with the bar location representing the mean of the data set and the standard deviation represented by error bars. Calculated *p*‐values are indicated using stars (*p < 0.05, **p < 0.01, ***p < 0.001, ****p < 0.0001). Welsh *t*‐tests were used to compare between two groups (assuming unequal standard variance within the data), while ANOVA with Bonferroni correction was utilized to compare between more than two groups. Survival data were obtained using the Kaplan–Meyer method with multiple group comparison correction (Bonferroni). Plots of survival data indicate median survival as calculated by Graphpad. Statistics for QPCR analysis relied on quantification by the delta–delta CT method followed by either *t*‐test or multiple comparison corrected ANOVA in Graphpad. For flow cytometry graphs, each data point represents an individual mouse (technical replicates) and each experiment was replicated fully at least once (biological replicates) with n > 3 mice (separated evenly by gender).

Statistics for TCR sequencing were supplied with the quantified data files transferred from adaptive because of their proprietary bioinformatics method for correction of PCR duplication errors (Carlson et al., 2013). R and the ggplot package were utilized to visualize measurements provided by adaptive such as productive clonality, Simpson clonality, distributed CDR3 length, clone productive frequency, and TCR beta gene usage. Simpson clonality measurements across multiple samples were compared and corrected using Dunn's post‐hoc correction. Standard deviations of transcript length or TCRB gene usage were testing using a two‐way *F* test. Productive clonality and TCRB gene usage were analyzed by comparing the mean productive clonality between top 20 clones, or TCRB gene productive frequency, per sample using two‐way ANOVA with Tukey correction for multiple comparisons.

## AUTHOR CONTRIBUTIONS

JMS, LZ, XW, NG, ED, AK, RD, and SH performed the experiments and collected the data. JMS, MD, and JG wrote and approved the manuscript. MD supervised the project and obtained funding. JMS completed data analysis and statistics.

## FUNDING INFORMATION

This work was supported by the NIH K08NS092895 grant (MD). JMS is partly supported by NIH/NINDS T32 NS105602.

## COMPETING INTERESTS

A US Provisional Patent application for GIFT‐7 has been made by JG prior to publication and is assigned to Emory University.

## Supporting information


Figure S1:

Figure S2:

Figure S3:

Figure S4:

Figure S5:

Figure S6:

## Data Availability

Sequencing data used for conclusions in this manuscript will be deposited into GEO repository upon publication of the manuscript. Code used for figure construction and analysis of TCR sequencing will be available upon the publication of this article. Any other relevant data involved in manuscript production was provided to the journal at time of submission and will be made available upon reasonable request to the corresponding author.
