# Supplementary figures and images for: GM‐CSF and IL‐7 fusion cytokine engineered tumor vaccine generates long‐term Th‐17 memory cells and increases overall survival in aged syngeneic mouse models of glioblastoma
Source: Aging Cell. 2023 May 11;22(7):e13864. doi: 10.1111/acel.13864 (PMC10352573; doi:10.1111/acel.13864)

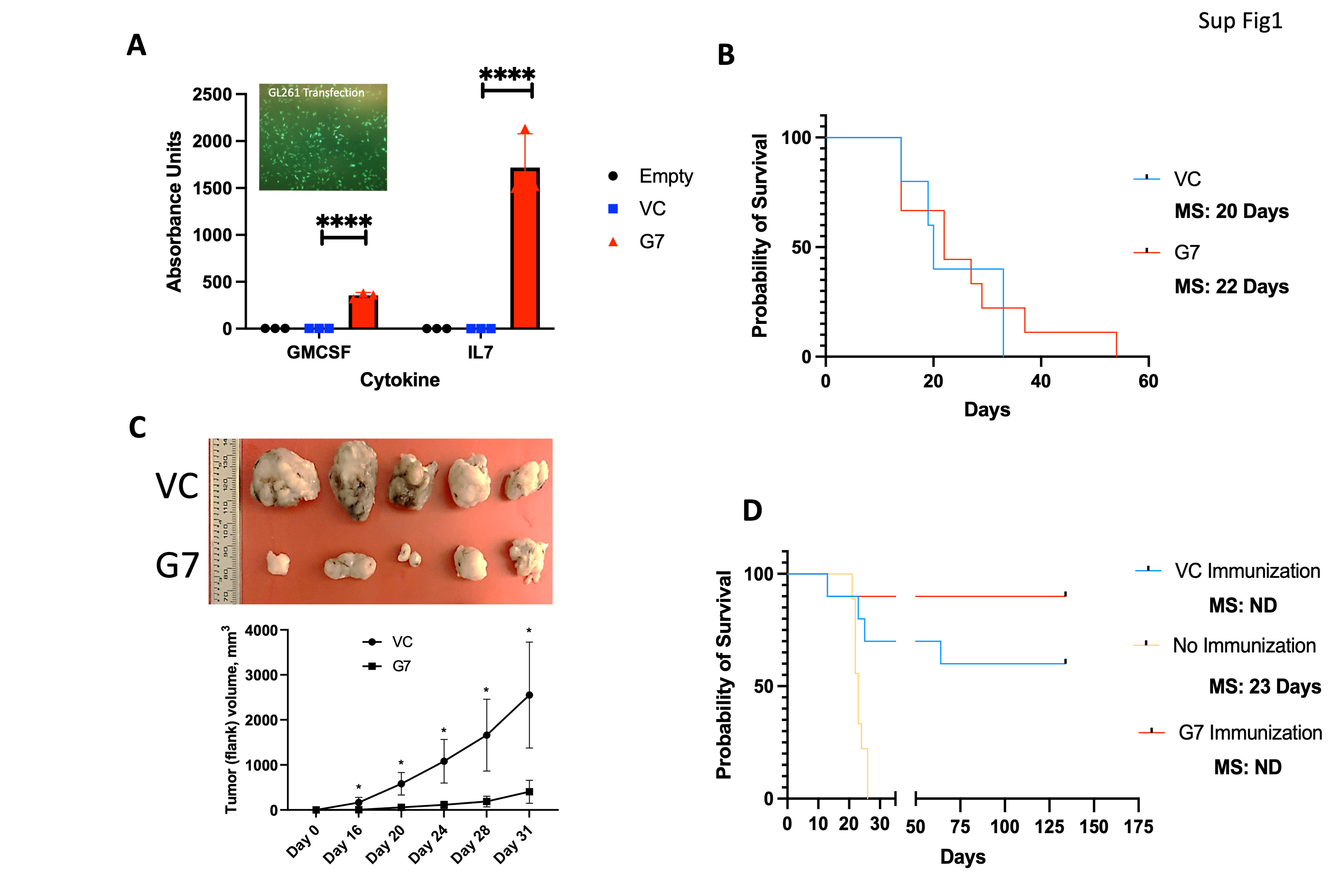

Supplement: Supplementary file 1 — Figure S1: [file ACEL-22-e13864-s003.tiff]

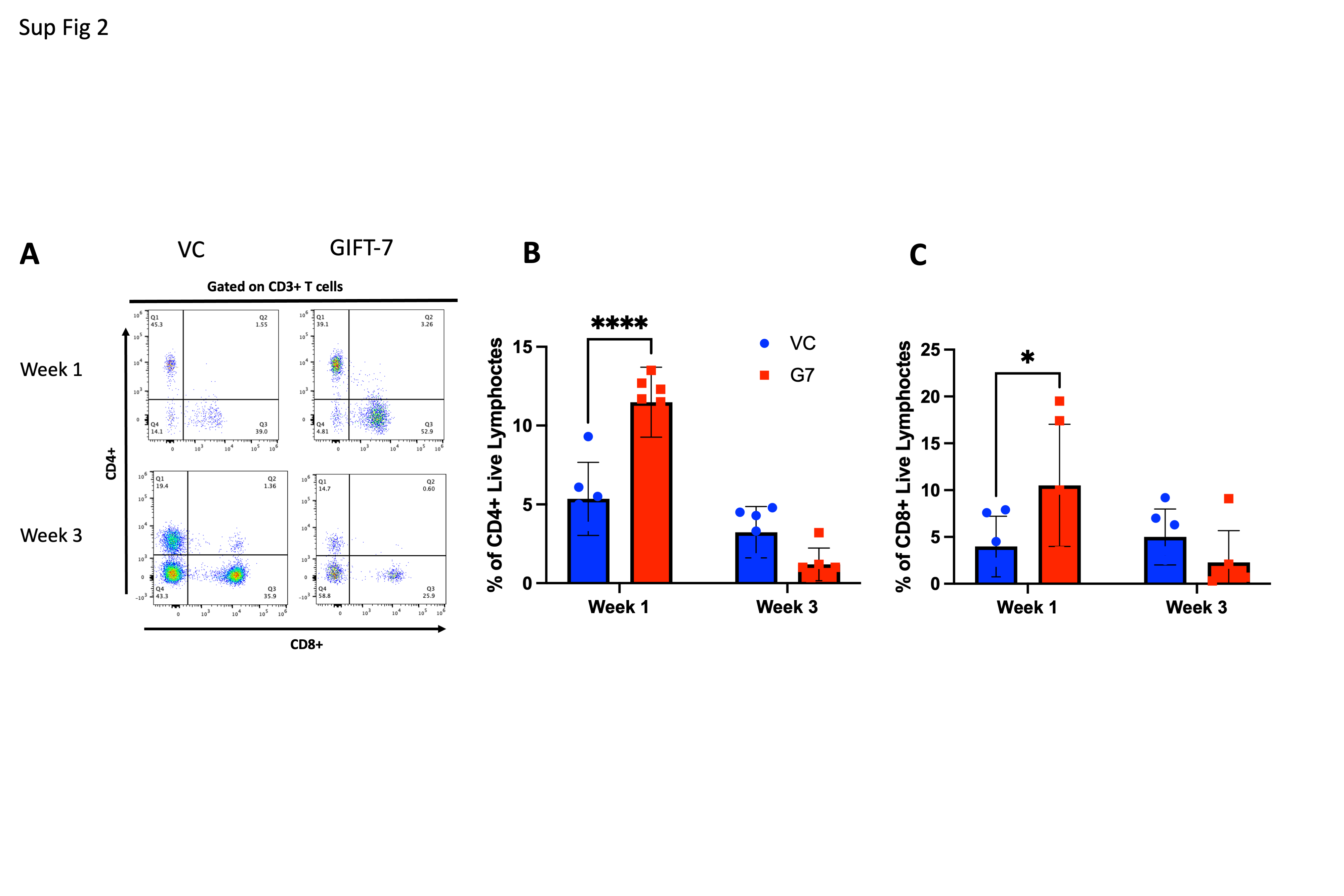

Supplement: Supplementary file 2 — Figure S2: [file ACEL-22-e13864-s006.tiff]

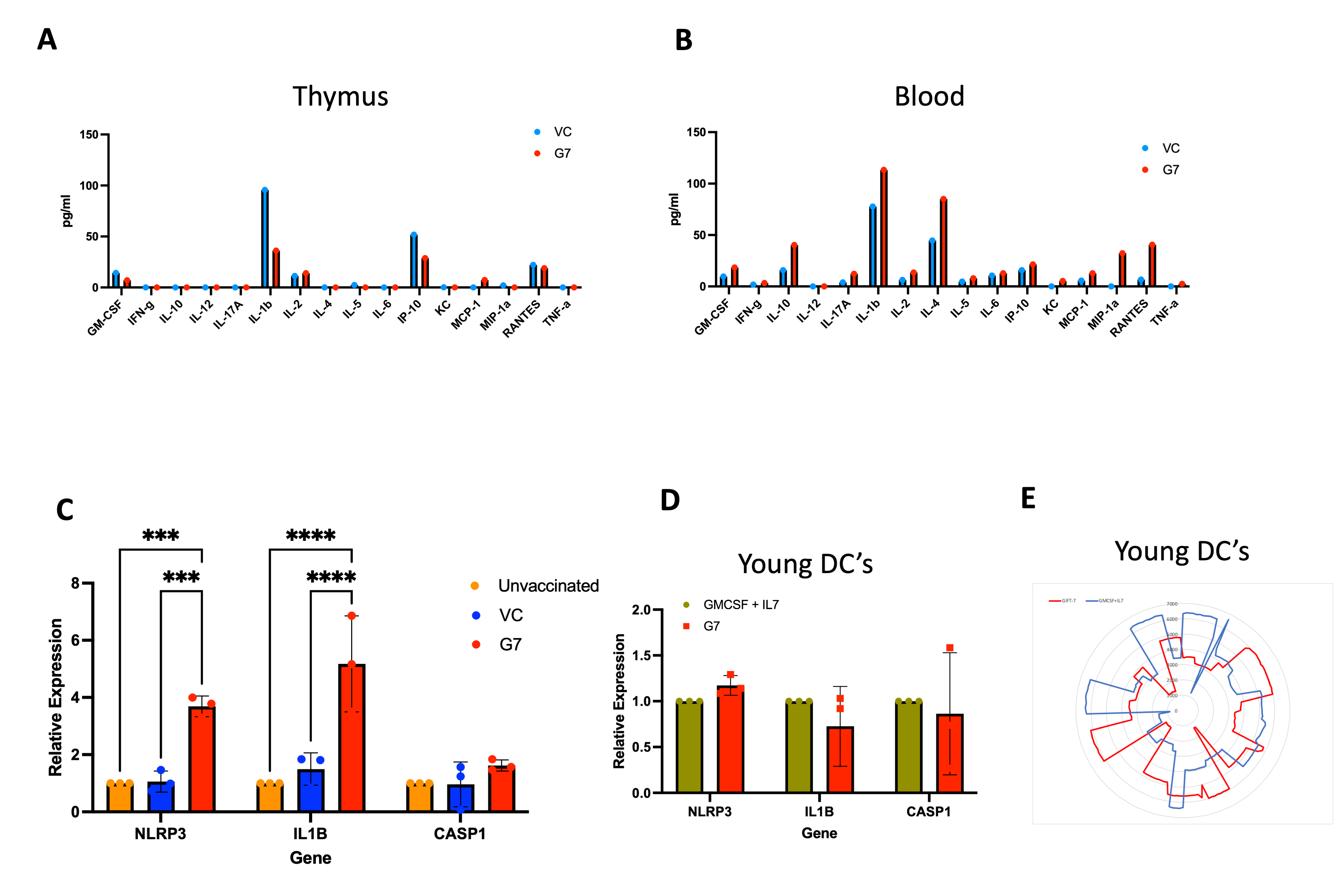

Supplement: Supplementary file 3 — Figure S3: [file ACEL-22-e13864-s001.tiff]

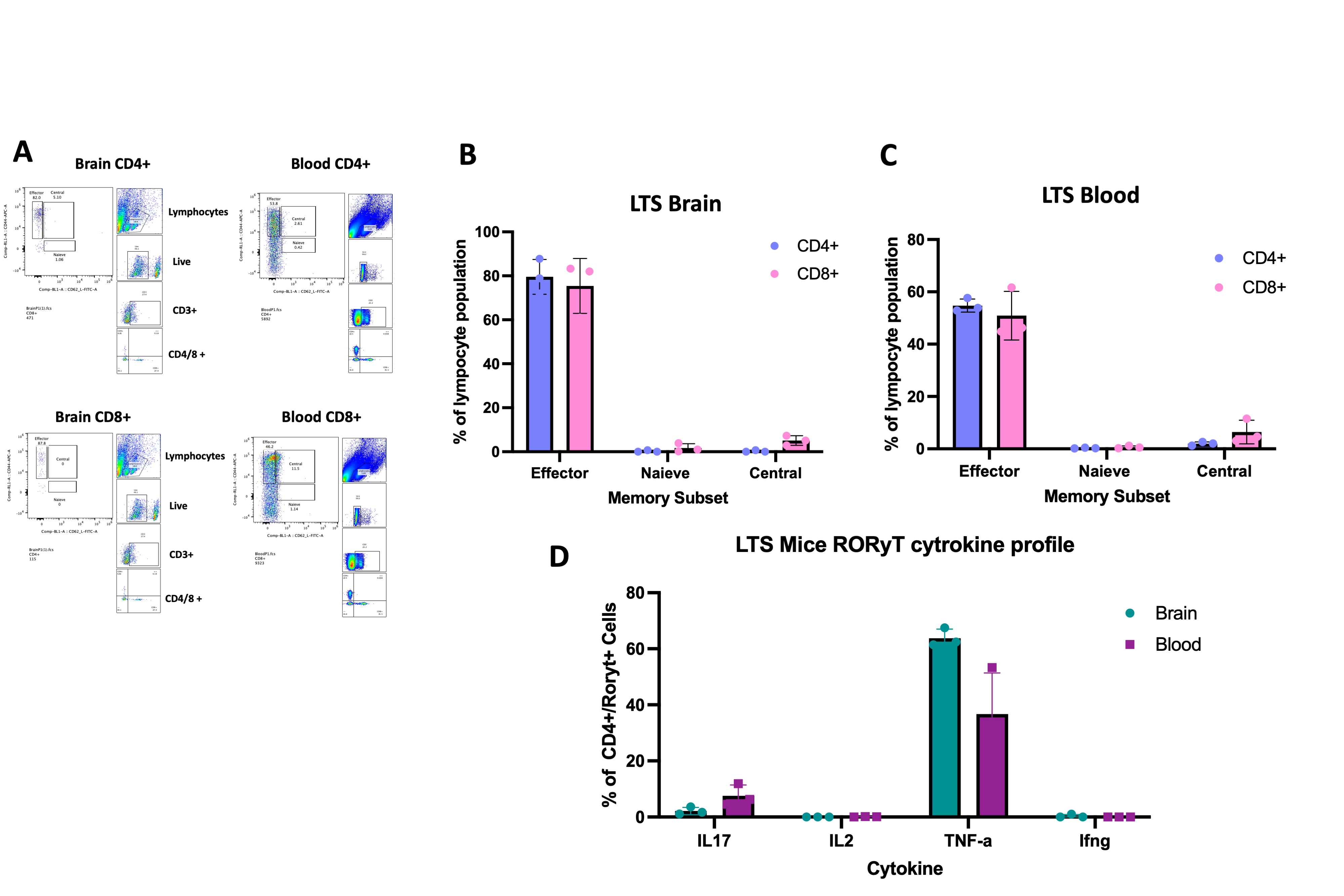

Supplement: Supplementary file 4 — Figure S4: [file ACEL-22-e13864-s004.tiff]

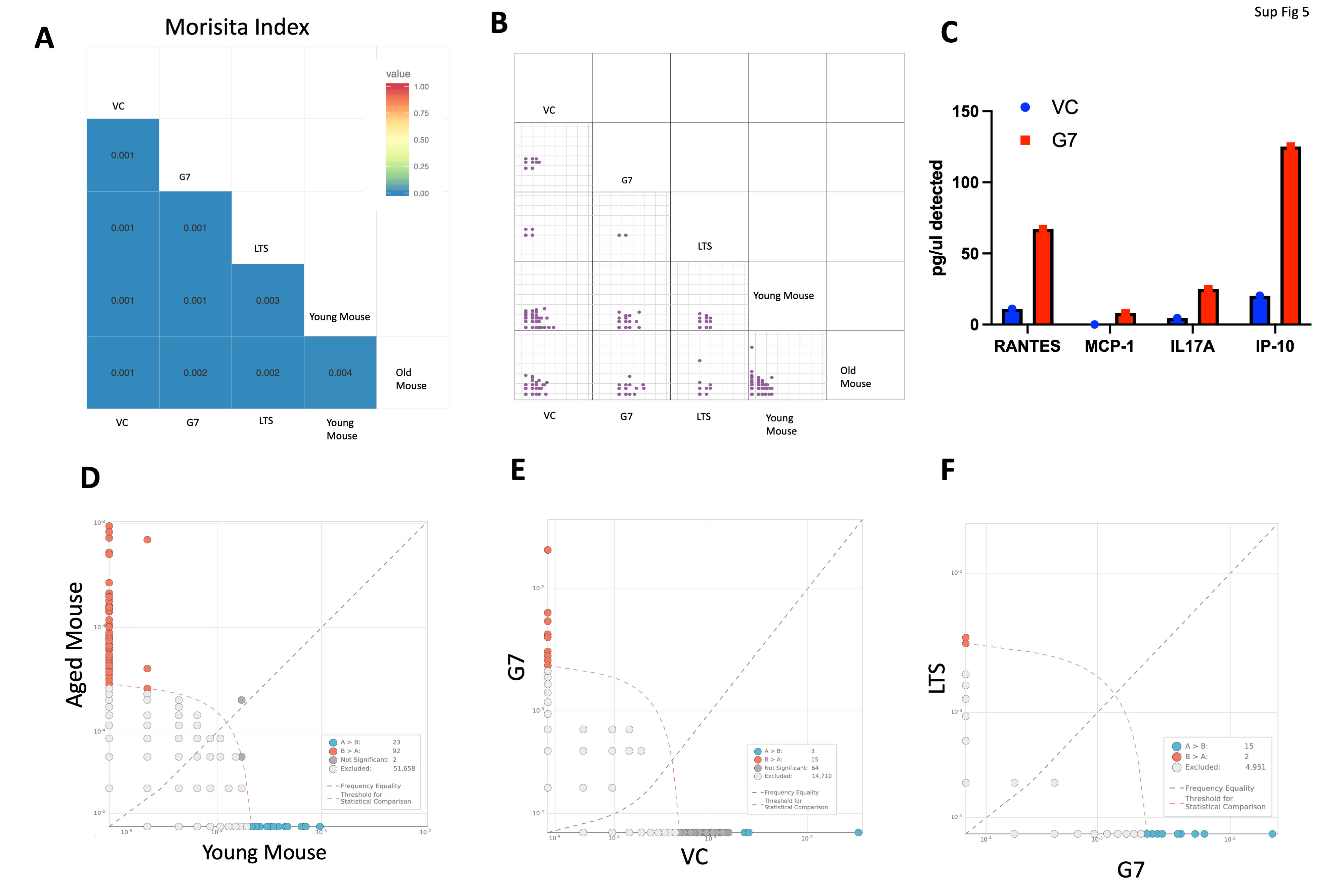

Supplement: Supplementary file 5 — Figure S5: [file ACEL-22-e13864-s002.tiff]

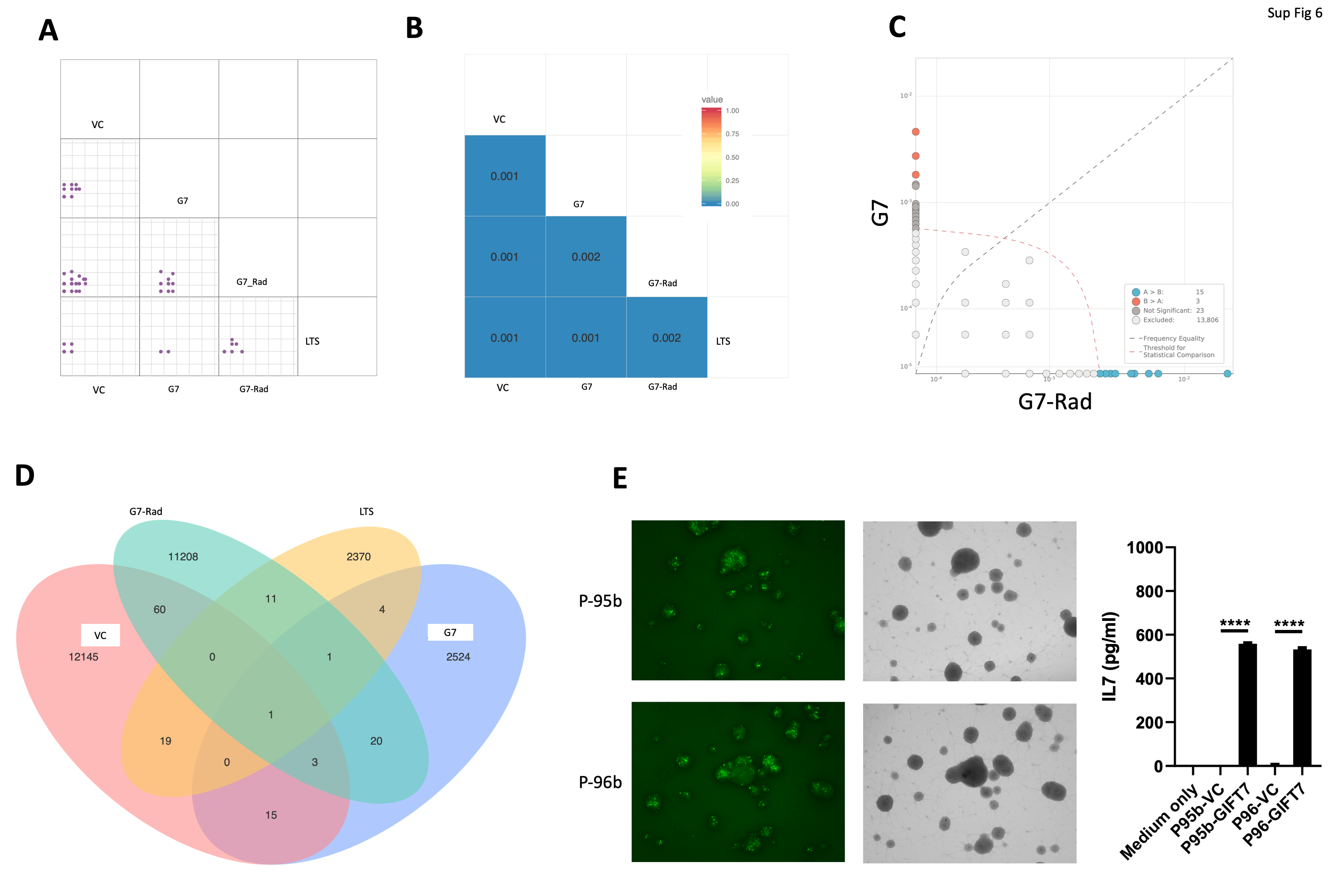

Supplement: Supplementary file 6 — Figure S6: [file ACEL-22-e13864-s005.tiff]
